# Deficiency of calcium/calmodulin-dependent serine protein kinase disrupts the excitatory-inhibitory balance of synapses by down-regulating GluN2B

**DOI:** 10.1038/s41380-018-0338-4

**Published:** 2019-01-04

**Authors:** Takuma Mori, Enas A. Kasem, Emi Suzuki-Kouyama, Xueshan Cao, Xue Li, Taiga Kurihara, Takeshi Uemura, Toru Yanagawa, Katsuhiko Tabuchi

**Affiliations:** 1 Department of Molecular and Cellular Physiology, Institute of Medicine, Academic Assembly, Shinshu University, Nagano, 390-8621 Japan; 2 Department of Zoology, Faculty of Science, Kafr Elsheikh University, Kafr Elsheihk, 33511 Egypt; 3 Institute for Biomedical Sciences, Interdisciplinary Cluster for Cutting Edge Research, Shinshu University, Nagano, 390-8621 Japan; 4 CREST, JST, Saitama, 332-0012 Japan; 5 Department of Oral and Maxillofacial Surgery, Faculty of Medicine, University of Tsukuba, Ibaraki, 305-8575 Japan; 6 PRESTO, JST, Saitama, 332-0012 Japan

**Keywords:** Physiology, Neuroscience

## Abstract

Calcium/calmodulin-dependent serine protein kinase (CASK) is a membrane-associated guanylate kinase (MAGUK) protein that is associated with neurodevelopmental disorders. CASK is thought to have both pre- and postsynaptic functions, but the mechanism and consequences of its functions in the brain have yet to be elucidated, because homozygous CASK-knockout (CASK-KO) mice die before brain maturation. Taking advantage of the X-chromosome inactivation (XCI) mechanism, here we examined the synaptic functions of CASK-KO neurons in acute brain slices of heterozygous CASK-KO female mice. We also analyzed CASK-knockdown (KD) neurons in acute brain slices generated by in utero electroporation. Both CASK-KO and CASK-KD neurons showed a disruption of the excitatory and inhibitory (E/I) balance. We further found that the expression level of the *N*-methyl-d-aspartate receptor subunit GluN2B was decreased in CASK-KD neurons and that overexpressing GluN2B rescued the disrupted E/I balance in CASK-KD neurons. These results suggest that the down-regulation of GluN2B may be involved in the mechanism of the disruption of synaptic E/I balance in CASK-deficient neurons.

## Introduction

Calcium/calmodulin-dependent serine protein kinase (CASK) is a membrane-associated guanylate kinase (MAGUK) protein that consists of N-terminal calcium/calmodulin kinase-like (CAM), LIN, PDZ, SH3, and C-terminal guanylate kinase (GK) domains [1–3]. CASK interacts with other synaptic proteins involved in cell adhesion, cytoskeletal organization, signal transduction, and/or gene transcription [4–12], and is thought to have both pre- and postsynaptic functions. As a postsynaptic role, CASK is translocated to the nucleus and functions as a co-activator of transcription regulatory factor T-brain-1 (TBR1), a T-box transcription factor, through binding at its GK domain [13]. The cooperation of CASK and TBR1 facilitates the transcription of T-element-containing genes, such as Reelin and the *N*-methyl-d-aspartate (NMDA) receptor subunit GluN2B [13–15].

Genomic variants of CASK have been linked to neurodevelopmental disorders [16], including mental retardation with or without nystagmus [17, 18], Otahara syndrome [19], infantile spasms [20, 21], mental retardation and microcephaly with pontine and cerebellar hypoplasia (MICPCH) [22, 23], and FG syndrome 4 [24, 25]. Variants that disrupt the entire CASK protein, such as deletions or stop mutations located in N-terminal regions, have been identified only in females [26]. CASK mutations may affect males more severely [27] because the human CASK gene is located on the X-chromosome (mapped to Xp11.4) [28, 29]. The complete loss of CASK is probably lethal, as observed in knockout (KO) mice [30]. Genes on the X-chromosome are subjected to X-chromosome inactivation (XCI) in female humans [31] and mice [32]. During early embryogenesis, one of the two alleles of the X-chromosome is randomly selected and inactivated by non-coding RNA called an X-inactive specific transcript. The inactivated and activated pair of X-chromosomes is transmitted to daughter cells after every cell division, resulting in a random mixture of cells with two different X genotypes throughout the body. Thus, both CASK-intact and CASK-deficient cells are likely to be distributed in a mosaic pattern in the brain of affected females. The mosaicism of CASK mutant neurons affects normal brain networks and may be responsible for the pathogenesis of neurological disorders.

The synaptic functions of CASK-KO mice were previously analyzed in dissociated cortical cultured neurons [30]. CASK-KO disrupts the balance between excitatory and inhibitory (E/I) synaptic functions, as indicated by an increase in the frequency of miniature excitatory postsynaptic currents (mEPSCs) and a decrease in the frequency of miniature inhibitory postsynaptic currents (mIPSCs).

Despite accumulating knowledge about CASK at the protein and cellular levels, its functions in the brain remain elusive. Here we examined the effect of CASK on synaptic function in acute brain slices, taking advantage of XCI and in utero electroporation. In patch-clamp electrophysiological analyses, both CASK-KO and CASK-KD pyramidal neurons in the forebrain replicated the disrupted E/I balance phenotype observed in cultured KO neurons. Since CASK-deficient neurons are distributed in a mosaic pattern in these tissues, we concluded that this effect was caused by a postsynaptic CASK deficiency. Rescue experiments in CASK-KD neurons showed that this disrupted E/I balance was due to the down-regulation of GluN2B transcription, which is normally promoted by the cooperation of TBR1 and CASK.

## Materials and methods

All animal procedures were approved by the Animal Care and the Use Committee of Shinshu University School of Medicine. Detailed procedures are described in the Supplementary Information.

### Animals

CASK-KO mice were obtained by crossing mice carrying floxed CASK (B6;129-Cask^tm1Sud^/J, JAX Stock #006382) [30] and ZP3-Cre (C57BL/6-Tg(Zp3-cre)93Knw/J [33], JAX Stock #003651).

### Plasmid construction

Short hairpin RNA (shRNA) constructs (see Supplementary Information for target sequences) were cloned into an L309 backbone vector [34]. The CASK, GluN2B, and tdTomato genes in eukaryotic expression vectors were driven by a CAG promoter.

### In utero electroporation

In utero electroporation was performed essentially as described previously [35, 36]. Briefly, pregnant Institute of Cancer Research mice at E15.5 were anesthetized, and the uterine horns were exposed. Approximately 1 μl of DNA solution was injected into the lateral ventricles of embryos. The embryos were subjected to five square electric pulses (35 V, 50 ms, 1 Hz) using an electroporator (CUY21E; NEPA Gene). The brains with abnormal morphology were excluded from the experiments.

### Electrophysiology

Slice recording was performed essentially as described previously [35]. P14–P18 mouse brains were cut into 350-μm-thick coronal sections. In the current clamp experiments, pyramidal neurons were patched with a glass pipette containing potassium-based intra-cellular solution (ICS) or cesium-based ICS. Miniature postsynaptic currents (mPSCs) were recorded in the presence of 1μM tetrodotoxin (Abcam). Membrane potential was held at −60mV for mEPSCs and 0 mV for mIPSCs. Evoked postsynaptic currents were triggered with a 0.1-ms current injection by a nichrome-wire electrode placed 100–50 μm from the soma of recorded neurons.

### Single-cell RT-PCR

Single-cell reverse transcription PCR (RT-PCR) was performed as described previously with modifications [35]. After the RT reaction, single-cell complementary DNA (DNA) was amplified by two rounds of semi-nested PCR with the primers listed in Supplementary Table 1. The animals were genotyped before recording and recorded cells were genotyped after the recording for blind experiments.

### Measurement of CASK and GluN2B mRNA levels in knockdown cultured neurons

Quantitative RT-PCR (qRT-PCR) was performed using the RNA isolated from cultured primary cortical neurons infected with shRNA-expressing lentivirus. The cells were harvested and lysed, and the total RNA was purified with Trizol reagent (Invitrogen). After cDNA synthesis, real-time PCR reactions (see Supplementary Table 1 for primers) were run and analyzed on a StepOnePlus system (Thermo Fisher).

### Sample size and statistical analysis

Samples sizes were determined based on established practice and on our previous experience in respective assays [30, 35, 37]. The number of independent samples (e.g., neurons) is indicated on the graphs and the number of animals is indicated in the figure legends. All values represent the average of independent experiments ± SEM. The variance among analyzed samples was similar. Statistical significance was determined by Student’s *t* test (for two groups) or one-way analysis of variance (ANOVA) followed by Bonferroni’s post hoc test (for multiple groups). Statistical analysis was performed with Prism 6.0 (Graphpad Software Inc.). Statistical significance is indicated by asterisks (*p < 0.05, **p < 0.01, ***p < 0.001).

## Results

### Heterozygous CASK-KO mice exhibit a hypomorphic phenotype and a mosaic distribution of CASK-deficient neurons in the brain due to XCI

**Fig. 1:**
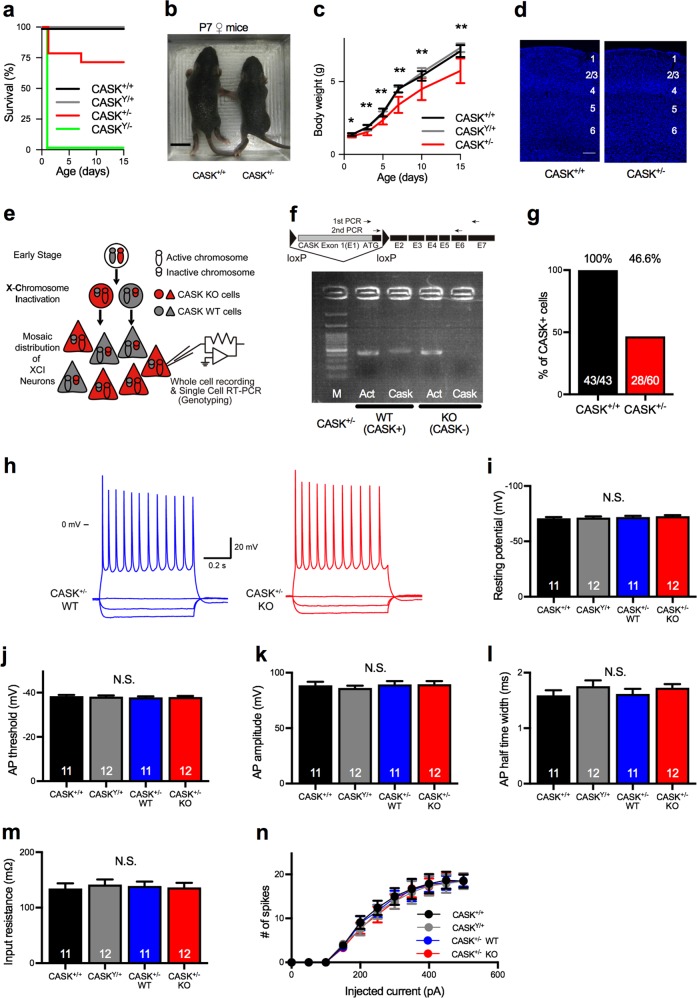
CASK is subjected to XCI in female mice. **a**. Survival rate of CASK-KO and WT mice. Approximately 25% of the CASK^+/−^ mice died within 15 days. Percentage of live animals of each genotype is shown. CASK^+/+^ (black, *n* = 12), CASK^Y/+^ (gray, *n* = 10), CASK^+/−^ (red, *n* = 14), and CASK^Y/−^ (green, *n* = 10). **b** Photographs of CASK^+/+^ (left) and CASK^+/−^ (right) female mice at 7 days of age. The CASK^+/−^ mice were smaller than the CASK^+/+^ mice. Scale bar represents 1 cm. **c** Growth curve of CASK-KO and WT mice. CASK^+/−^ mice (*n* = 10) were smaller than CASK^+/+^ (*n* = 10) and CASK^Y/+^ (*n* = 12) mice. **d** DAPI staining of the somatosensory cortex of 5-week-old CASK^+/+^ (left) and CASK^+/−^ (right) mice. Numbers indicate cortical layers. Scale bar: 100 µm. **e** Schematic illustration of XCI and single-cell genotyping in heterozygous CASK-KO mice. In the progenitor cells of female heterozygous CASK-KO mice, one of the X chromosomes is randomly selected and inactivated by XCI early in development. The XCI pattern is transferred to their daughter cells during cell division, resulting in a mosaic distribution pattern of the CASK WT and CASK-KO cells in tissues. In adult brain slices, the genotypes and physiological functions of neurons were examined by patch-clamp recording followed by single-cell RT-PCR. **f** Agarose gel electrophoresis image of single-cell RT-PCR. Positions of the primer sets for the RT-PCR of CASK are shown above. Note that the CASK band is absent in the right-most lane (KO; CASK^−^). M, 100-bp DNA ladder; Act, β-actin PCR product; Cask, CASK PCR product. **g** Percentage of CASK-positive cells. CASK expression was detected in about half of the cells in the CASK^+/−^ brains. CASK^+/+^, *n* = 6 animals; and CASK^+/−^, *n* = 10 animals. Numbers on bars are the CASK-positive cells/total cells analyzed. **h** Representative traces of the membrane potential of CASK-positive (WT, left, blue traces) and CASK-negative (KO, right, red traces) neurons from a CASK^+/−^ mouse. The CASK genotypes of the recorded neurons of CASK^+/−^ mice were determined by single-cell RT-PCR. Scale bars represent 20 mV (vertical axis) and 0.2 s (horizontal axis). 0 mV indicates the potential at the ground. **i**–**n** Resting membrane potential (**i**), AP threshold (**j**), AP amplitude (**k**), AP half-time width (**l**), input resistance (**m**), and input (injected current)/output (spike number) curve (**n**) of WT (CASK^+/+^, CASK^Y/+^, CASK^+/−^ WT) and CASK^+/−^ KO neurons (animal numbers; CASK^+/+^ female *n* = 3, CASK^Y/+^ male *n* = 3, CASK^+/−^ female *n* = 7). Numbers on bars are the numbers of cells analyzed (**i**–**m**). *p < 0.05, **p < 0.01, as examined by ANOVA and Bonferroni’s post hoc test. N.S. not significant

To examine the physiological role of CASK, we studied the effect of CASK-KO in adult mouse brain. As described previously [30], all CASK-KO mice (CASK^Y/−^) died within 24 h after birth. In contrast, the survival and growth of heterozygous female KO mice (CASK^+/−^) varied among individuals. Approximately 75% of the CASK^+/−^ mice were viable at P15 (Fig. 1a), with 63.5–102.9% of the mean wild-type (WT) body weight (Fig. 1b, c). The cerebellar cortex was smaller in the CASK^+/−^ mice compared to WT (Supplementary Figure 1a), but its overall structure, including its laminar arrangement, was normal (Fig. 1d and Supplementary Figure 1b). We did not observe apparent spontaneous seizures, but found a reduction of seizure threshold in CASK^+/−^ mice by pentylenetetrazol injection (Supplementary Figure 1c and 1d).

We speculated that the variable survival and body weight of the CASK^+/−^ mice was due to XCI. Thus, we examined the CASK genotype in single neurons in acute brain slices of CASK^+/−^ mice by single-cell RT-PCR, using cytosol from pyramidal neurons extracted through patch pipettes [35] (Fig. 1e, f). We found that approximately half (46.6 %) of these neurons expressed CASK messenger RNA (mRNA) while half did not (Fig. 1g), suggesting that CASK was subjected to XCI. The same method was used hereafter to determine the CASK genotype in neurons recorded by patch-clamp electrophysiology in CASK^+/−^ mice.

CASK is known to affect the properties of ion channels, including calcium (e.g., Cav1.2) [38, 39], inward rectifier potassium (Kir2) [40], and voltage-gated sodium (NaV 1.5) [41] channels. To investigate whether CASK deletion affected the function of ion channels in neocortical pyramidal neurons, we examined the membrane properties and excitability in CASK^+/−^-KO neurons by whole-cell recording in the current clamp mode (Fig. 1h). In this experiment, we did not detect changes in any of the parameters we tested, including the resting potential, threshold of AP, amplitude of AP, half-time width of AP, input resistance, or input–output relationship between the injected current amount and spike numbers (Fig. 1i–n), suggesting that the CASK deletion did not affect the intrinsic excitability in cortical pyramidal neurons.

### Postsynaptic CASK deficiency during development disrupts the E/I balance in synaptic transmission in heterozygous CASK-KO mice

**Fig. 2:**
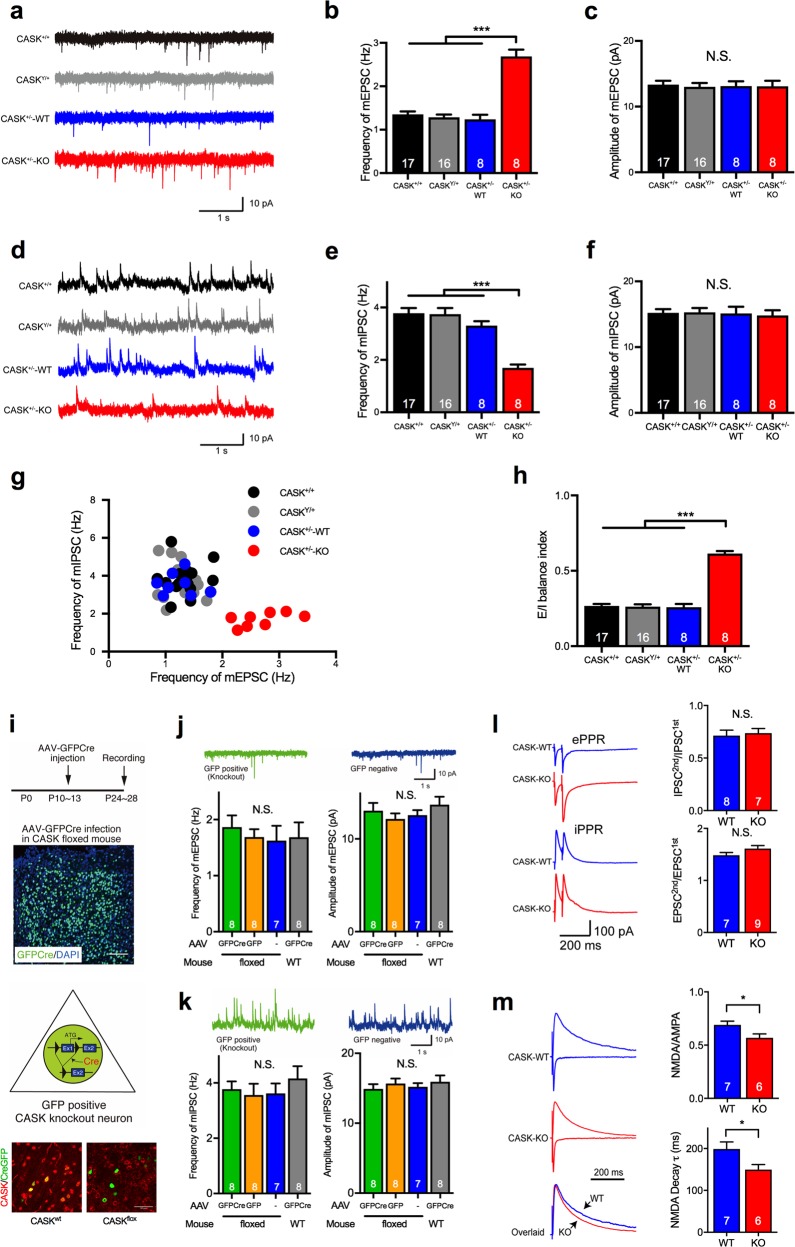
CASK deficiency disrupts the E/I balance and NMDA receptor-mediated synaptic function in a developmental mechanism. **a** Representative traces of miniature excitatory postsynaptic currents (mEPSCs) in neurons with four different genotypes. The CASK genotype of the recorded neurons from CASK^+/−^ mice was determined by single-cell RT-PCR. Scale bars represent 10 pA (vertical axis) and 1 s (horizontal axis). **b**, **c** Frequency (**b**) and amplitude (**c**) of the mEPSCs in neurons with four different genotypes. The frequency of mEPSCs in CASK-deficient neurons (CASK^+/−^-KO) was increased. Numbers on bars represent the number of cells recorded (animal numbers; CASK^+/+^ female *n* = 3, CASK^Y/+^ male *n* = 3, CASK^+/−^ female *n* = 7). **d** Representative traces of the mIPSCs in neurons with four different genotypes. The CASK genotype of the recorded neurons from CASK^+/−^ mice was determined by single-cell RT-PCR. Scale bars represent 10pA (vertical axis) and 1 s (horizontal axis). **e**, **f** Frequency (**e**) and amplitude (**f**) of the mIPSCs in neurons with four different genotypes. The frequency of the mIPSCs in CASK-deficient neurons (CASK^+/−^-KO) was decreased. Numbers on bars represent the number of cells recorded (animal numbers; CASK^+/+^ female *n* = 3, CASK^Y/+^ male *n* = 3, CASK^+/−^ female *n* = 7). **g** Scatter plot of the frequency of mEPSCs versus mIPSCs in neurons with four different genotypes. Each dot represents a single cell. CASK-deficient cells (CASK^+/−^-KO) showed a different pattern from those of the other genotypes. **h** E/I balance index for each genotype. The E/I balance of CASK-deficient cells (CASK^+/−^-KO) was shifted toward excitatory dominance. Numbers on bars represent the number of cells recorded. **i** AAV-Cre-mediated conditional knockout of CASK. The timings of AAV injection and recording (top), a histological image of the AAV-injected area (middle), and a schematic illustration of the conditional deletion of CASK in a cell and confocal images for CASK immunostaning (red) in AAV-CreGFP (green) infected brains in wild-type and CASK-floxed mice (bottom) are shown. Note that the CASK and GFP double-positive neurons are missing in CASK-floxed mice. Scale bars are 100 μm (middle) and 50 μm (bottom). **j**, **k** Frequency and amplitude of mEPSCs (**l**) and mIPSCs (**m**) were unaltered in CASK conditional KO neurons (green). Numbers on bars represent the number of cells recorded (animal numbers; floxed CASK + GFP-Cre *n* = 4, floxed CASK + GFP *n* = 3, floxed CASK *n* = 3, WT + GFP-Cre *n* = 3). **l** Paired-pulse ratios of evoked excitatory and inhibitory postsynaptic contents (ePPR and iPPR) were unaltered in CASK^+/−^-KO neurons. Representative traces (left) and summary graphs (right) are shown. Scale bars represent 100pA (vertical axis) and 200 ms (horizontal axis). Numbers on bars represent the number of cells recorded (animal numbers; CASK^+/−^ female *n* = 9). **m** Representative traces (left) and summary graphs (right) of the NMDA/AMPA ratio and NMDA decay time constant of WT and CASK^+/−^-KO neurons. The NMDA/AMPA ratio and decay time constant of the NMDA current were decreased in CASK^+/−^-KO neurons. Scale bar represents 200 ms. Numbers on bars represent the number of cells recorded (animal numbers; CASK^+/−^ female *n* = 8). Statistical significance was determined by ANOVA and Bonferroni’s post hoc test. *p < 0.05, ***p < 0.001. N.S. not significant

Next, to examine the effect of CASK deficiency on synaptic function, we studied the spontaneous mPSCs in CASK^+/−^ and WT mice. We recorded both the mEPSCs and mIPSCs from each pyramidal neuron in layer 2/3 of the somatosensory cortical slices in CASK^+/+^, CASK^Y/+^, and CASK^+/−^ mice. We identified the genotypes of the postsynaptic neurons by single-cell RT-PCR after recording as described above. We found that the frequency but not the amplitude of the mEPSCs was significantly increased in the CASK^+/−^-KO neurons in CASK^+/−^ mice (Fig. 2a–c). In contrast, the frequency but not the amplitude of the mIPSCs was decreased in the CASK^+/−^-KO neurons in CASK^+/−^ mice (Fig. 2d–f). A scatter plot revealed that the distribution of the frequency of mEPSCs versus mIPSCs in CASK^+/−^-KO neurons was different from that in the other genotypes (Fig. 2g). The E/I balance index in CASK^+/−^-KO neurons (0.615 ± 0.016) was significantly higher than that in the WT genotypes (CASK^+/+^ = 0.267 ± 0.013, CASK^Y/+^ = 0.262 ± 0.015, CASK^+/−^-WT neurons = 0.259 ± 0.021, *p* < 0.001, ANOVA, Bonferroni’s post hoc test; Fig. 2h). Similar phenotypes were also observed in the pyramidal neurons of the CA1 region of the hippocampus (Supplementary Figure 1). In CASK^+/−^ mice, approximately half of the presynaptic inputs onto recording neurons should be CASK-positive and the other half CASK-negative. The genotype-dependent phenotype of the recording neurons suggested that these effects were caused by CASK deficiency in the postsynaptic neurons.

Next, to address whether the disrupted E/I balance phenotype in CASK^+/−^-KO neurons was due to the absence of CASK during neural development or after synapse formation, we knocked out CASK in the adult cerebral cortex by AAV-mediated Cre recombination. We injected AAV-GFP-Cre or AAV-GFP into the somatosensory cortex of CASK-floxed mice at P10–13 and analyzed mPSCs in acute brain slices at P24–28 (Fig. 2i). We recorded from the GFP-Cre-positive cells, in which CASK was knocked out by Cre recombination. The adult CASK-KO neurons exhibited unaltered mPSCs (Fig. 2j, k), suggesting that the disrupted E/I balance was caused by the CASK deficiency during neural development.

### CASK deficiency impairs NMDA receptor-mediated synaptic function

Alterations in the frequency but not in the amplitude of mPSCs indicated that presynaptic functions projecting to the CASK^+/−^-KO neurons might have been impaired. To examine the release probability of presynaptic inputs, we analyzed the paired-pulse ratio (PPR) of the E/I neurotransmission evoked by electrical stimulation. We found that the PPRs of both E/I inputs were unaltered in the CASK^+/−^-KO neurons (Fig. 2l), indicating that CASK deficiency did not affect the release probability of synaptic inputs, but instead might have affected the number of functional synapses. We further investigated the NMDA receptor function by measuring the evoked NMDA currents recorded at a holding potential of +40 mV. We found that the NMDA/AMPA (α-amino-3-hydroxy-5-methyl-4-isoxazolepropionic acid ratio and NMDA decay time constant were decreased in the CASK^+/−^-KO neurons (Fig. 2m).

### Postsynaptic CASK KD disrupts the E/I balance of synaptic transmission and downregulates the expression of GluN2B

To investigate the mechanism by which CASK regulates the E/I balance of synapses during neural development, we knocked down CASK specifically in a population of pyramidal neurons in layer 2/3 of the somatosensory cortex using in utero electroporation. The CASK mRNA level in the KD neurons was decreased to 13.5% of that found in control virus-infected neurons (Supplementary Figure 3a). The CASK-KD neurons showed normal migration in the six-layer cortical structure (Supplementary Figure 3b) and unaltered spine density (Supplementary Figure 4). CASK-KD also did not affect the membrane properties or the intrinsic excitability of neurons (Supplementary Figure 5).

**Fig. 3:**
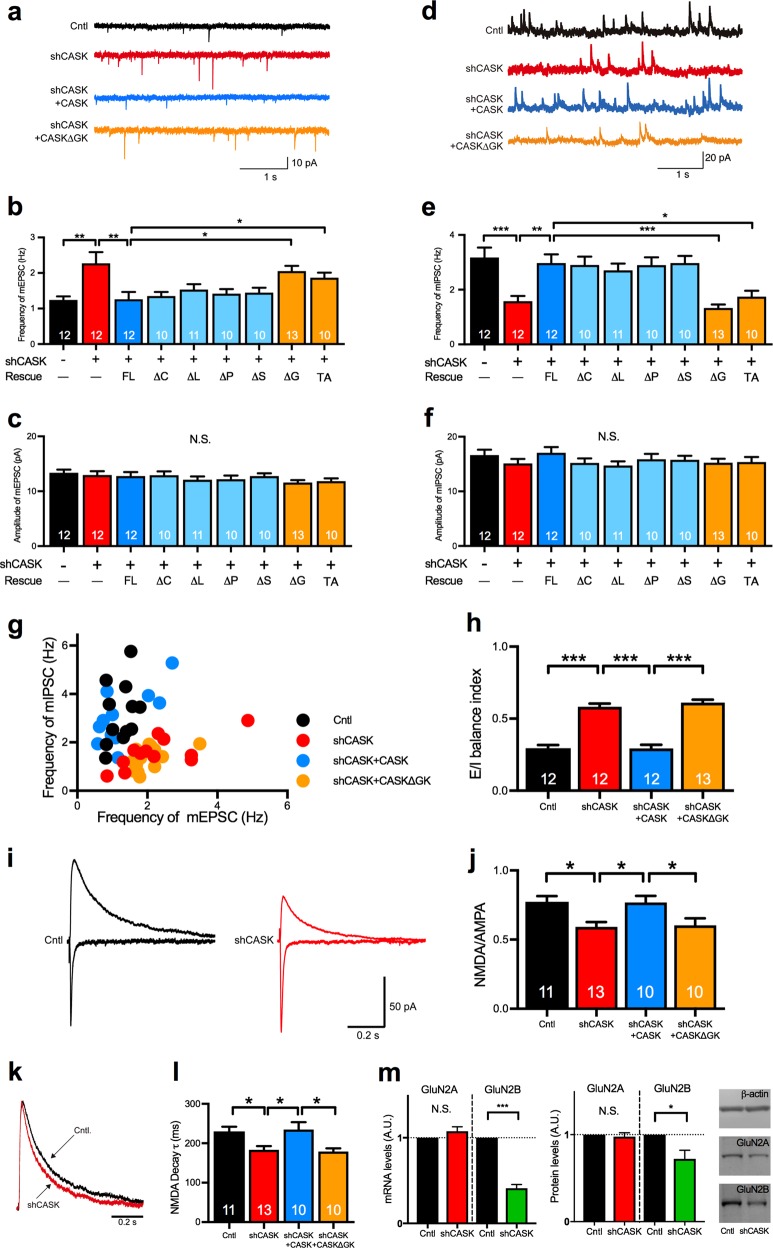
The guanylate kinase domain of CASK is responsible for the disrupted E/I balance and NMDA receptor function in CASK-KD neurons. **a**–**h** Miniature postsynaptic currents of control and CASK-KD neurons co-transfected with or without various rescue constructs in layer 2/3 of the somatosensory cortex. Representative traces (**a**) and graphs of the frequency (**b**) and amplitude (**c**) of mEPSCs. Representative traces (**d**), summary graphs of the frequency (**e**), and amplitude (**f**) of mIPSCs. FL, full-length; ΔC, CAM domain deleted; ΔL, LIN domain deleted; ΔP, PDZ domain deleted; ΔS, SH3 domain deleted; ΔG and ΔGK, GK domain deleted; TA, T704A mutant of CASK. The CASK ΔG or TA constructs failed to rescue the increased frequency of mEPSCs (**b**) or the decreased frequency of mIPSCs (**e**) in CASK-KD neurons. Scale bars represent 10 pA (vertical axis) (**a**) and 20 pA (vertical axis) (**d**) and 1 s (horizontal axis) (**a**, **d**) (animal numbers; *n* = 3 in each condition). **g** Distribution of the frequency of mEPSCs versus mIPSCs for neurons of four genotypes. Each dot represents a single cell. **h** E/I balance index for neurons with each KD. **i** Representative traces of evoked AMPA (lower trace) and NMDA (upper trace) receptor-medicated synaptic currents in control and CASK-KD neurons. Scale bars represent 50 pA (vertical axis) and 0.2 s (horizontal axis). **j** Graph of the NMDA/AMPA ratio in control, CASK-KD, and CASK-KD + rescue vector-transfected neurons (animal numbers; *n* = 3 in each condition). **k** Normalized traces of NMDA receptor-mediated currents recorded from control and CASK-KD neurons were superimposed. Scale bar represents 0.2 s. **l** Weighted decay time constant (*τ*) of the NMDA receptor-mediated current in control, CASK-KD, and CASK-KD + rescue vector-transfected neurons. The decay time constant of the NMDA current was decreased in CASK-KD neurons, and was not rescued by CASKΔGK co-transfection. **m** The mRNA (left) and protein (middle) levels of GluN2A and GluN2B in control and CASK-KD neurons examined by qRT-PCR and immunoblot, respectively. Representative images (right) of immunoblot bands of β-actin, GluN2A, and GluN2B obtained from control and CASK-KD neurons (sample numbers; Cntl *n* = 3, shCASK *n* = 3 in mRNA; Cntl *n* = 5, shCASK *n* = 5 in protein). Statistical significance was determined by unpaired *t* test (**m**) or by ANOVA and Bonferroni’s post-hoc test (**b**, **c**, **e**, **f**, **h**, **j**, and **l**). *p < 0.05, **p < 0.01, and ***p < 0.001. Numbers on bars are the number of cells analyzed. N.S. not significant

We next measured the mEPSCs and mIPSCs in the CASK-KD neurons. Consistent with the results of CASK^+/−^-KO neurons, the CASK-KD neurons had mEPSCs with an increased frequency but unaltered amplitude (Fig. 3a–c), and mIPSCs with a decreased frequency and unaltered amplitude (Fig. 3d–f). The scatter plot of the frequency of mEPSCs versus mIPSCs was different for the CASK-KD neurons compared to controls (Fig. 3g). The E/I balance index of the CASK-KD neurons (0.583 ± 0.022; Fig. 3h) was greater than that of controls (0.295 ± 0.022). Like CASK-KO, CASK-KD showed no change in either the excitatory or the inhibitory PPRs (Supplementary Figure 6).

CASK contains CAM, LIN, PDZ, SH3, and GK domains. To investigate which domain of CASK is responsible for the synaptic phenotype, we performed rescue experiments in which we co-expressed deletion mutants of CASK lacking each domain in CASK-KD neurons (Supplementary Figure 7). The co-expression of CASK lacking the CAM (ΔC), LIN (ΔL), PDZ (ΔP), or SH3 (ΔS) domain, as well as CASK full-length (FL), restored the frequency of both the mEPSCs and mIPSCs to the control levels (Fig. 3a–f). However, CASK lacking the GK domain (ΔG) failed to rescue the altered frequencies of mEPSCs/mIPSCs and the disrupted E/I balance (Fig. 3a–h).

Consistent with the results in CASK-KO neurons, CASK-KD impaired the NMDA receptor function. The NMDA/AMPA ratio and decay time constant were decreased in CASK-KD neurons (Fig. 3i–l), and these phenotypes were not rescued by co-expressing the CASK ΔG construct (Fig. 3i–l). A shortened decay time constant of the NMDA receptor-mediated current implies that the composition of NMDA receptor subunits is altered [42]. We therefore examined the expression levels of GluN2A and GluN2B in CASK-KD cultured neurons and found that the GluN2B expression was selectively decreased (Fig. 3m).

The GluN2B expression is known to be regulated by T-box transcription factor TBR1 bound to the GK domain of CASK [15]. Indeed, we detected TBR1 immunoreactivity mostly in the layer 2/3 neurons of P14 mice (Supplementary Figure 8). To examine whether the synaptic phenotype of CASK-deficient neurons was related to the CASK/TBR1 interaction, we co-transfected them with CASK containing a T704A mutation, which interferes with CASK’s binding to TBR1, in the CASK-KD neurons and analyzed mPSCs [43–45]. Like the CASK ΔG mutant, the CASK-T704A mutant failed to rescue the aberrant frequencies of mEPSCs and mIPSCs (Fig. 3b, e).

### GluN2B-KD replicates the disrupted E/I balance of synaptic transmission

**Fig. 4:**
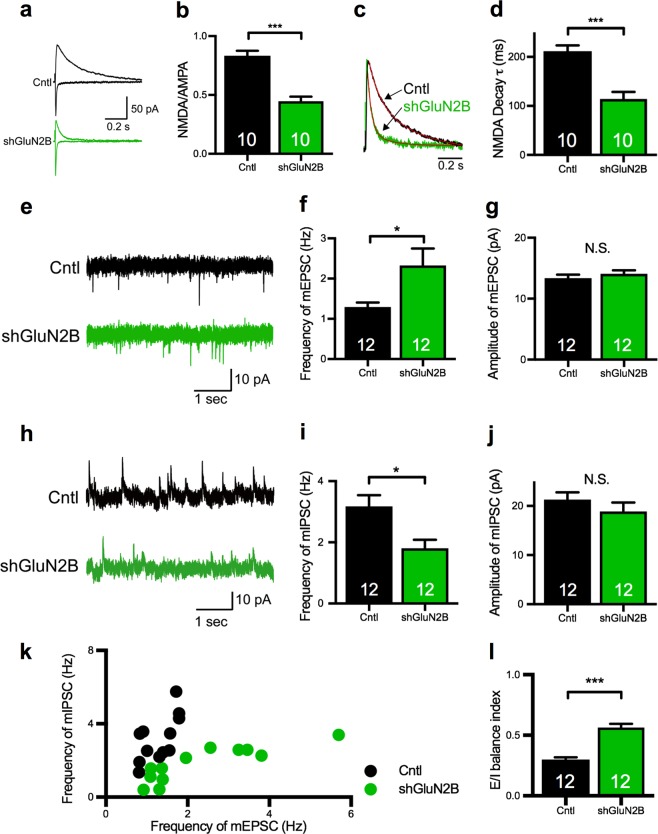
GluN2B-KD disrupts the E/I balance in pyramidal neurons in layer 2/3 of the somatosensory cortex. **a** Representative traces of evoked AMPA (lower trace) and NMDA (upper trace) receptor-mediated synaptic currents in control (black) and shGluN2B-transfected (green) neurons. Scale bars represent 50pA (vertical axis) and 0.2 s (horizontal axis). **b** NMDA/AMPA ratio in control and shGluN2B-transfected neurons. The NMDA/AMPA ratio was significantly decreased in the shGluN2B-transfected neurons (animal numbers; Cntl *n* = 3, shGluN2B *n* = 3). **c** Normalized sample traces of NMDA receptor-mediated synaptic currents recorded from control and shGluN2B-transfected neurons. Fitted double exponential curves are shown in red. Scale bar represents 0.2 s. **d** Weighted decay time constant (*τ*) of the NMDA receptor-mediated current in control and shGluN2B-transfected neurons. The decay time constant (*τ*) in shGluN2B-transfected neurons was decreased compared to control neurons (animal numbers; Cntl *n* = 3, shGluN2B *n* = 3). **e** Representative traces of the mEPSC in control (upper) and shGluN2B-transfected (lower) neurons. Scale bars represent 10pA (vertical axis) and 1 s (horizontal axis). **f**–**g** Frequency (**f**) and amplitude (**g**) of the mEPSC in control and shGluN2B-transfected neurons. The frequency of the mEPSC was increased by GluN2B-KD (animal numbers; Cntl *n* = 3, shGluN2B *n* = 3). **h** Representative traces of the mIPSC in control (upper) and shGluN2B-transfected (lower) neurons. Scale bars represent 10pA (vertical axis) and 1 s (horizontal axis). **i**–**j** Frequency (**i**) and amplitude (**j**) of the mIPSC in control and shGluN2B-transfected neurons. The frequency of the mIPSC was decreased by GluN2B-KD (animal numbers; Cntl *n* = 3, shGluN2B *n* = 3). **k** Scatter plot of the frequency of mEPSCs versus mIPSCs in control and shGluN2B-transfected neurons. Each dot represents a single cell. The control and shGluN2B-transfected neurons showed different distributions. **l** E/I balance index of control and shGluN2B-transfected neurons. Statistical significance was determined by unpaired *t*test. *p < 0.05, ***p < 0.001. Numbers on bars are the number of cells analyzed. N.S. not significant

Next, we examined the effects of GluN2B deficiency on synapses using GluN2B-KD and compared them to those of CASK-KD. We confirmed that our shRNA construct for GluN2B (shGluN2B) [46] suppressed the expression of GluN2B mRNA to 6.6% of the level of control construct-transfected neurons (Supplementary Figure 9a). Introducing shGluN2B into the pyramidal neurons in layer 2/3 of the somatosensory cortex by in utero electroporation did not affect the layer formation of the brain or the migration of KD neurons (Supplementary Figure 9b). Reflecting the loss of GluN2B function, the NMDA/AMPA ratio was decreased (Fig. 4a, b) and the decay time constant of the NMDA current was shortened in the GluN2B-KD neurons (Fig. 4c, d). As observed in CASK-KD neurons, the GluN2B-KD neurons had mEPSCs of increased frequency, but not amplitude (Fig. 4e–g). Likewise, the frequency but not the amplitude of the mIPSCs was decreased in the GluN2B-KD neurons (Fig. 4h–j). The E/I relationship was also different between the GluN2B-KD and control neurons (Fig. 4k). The E/I balance index in GluN2B-KD neurons was shifted toward excitatory dominance (Fig. 4l). The PPR was unchanged in GluN2B-KD neurons (Supplementary Figure 10), suggesting that the presynaptic release machinery was unaffected.

### GluN2B rescues the disrupted E/I balance caused by CASK deficiency

**Fig. 5:**
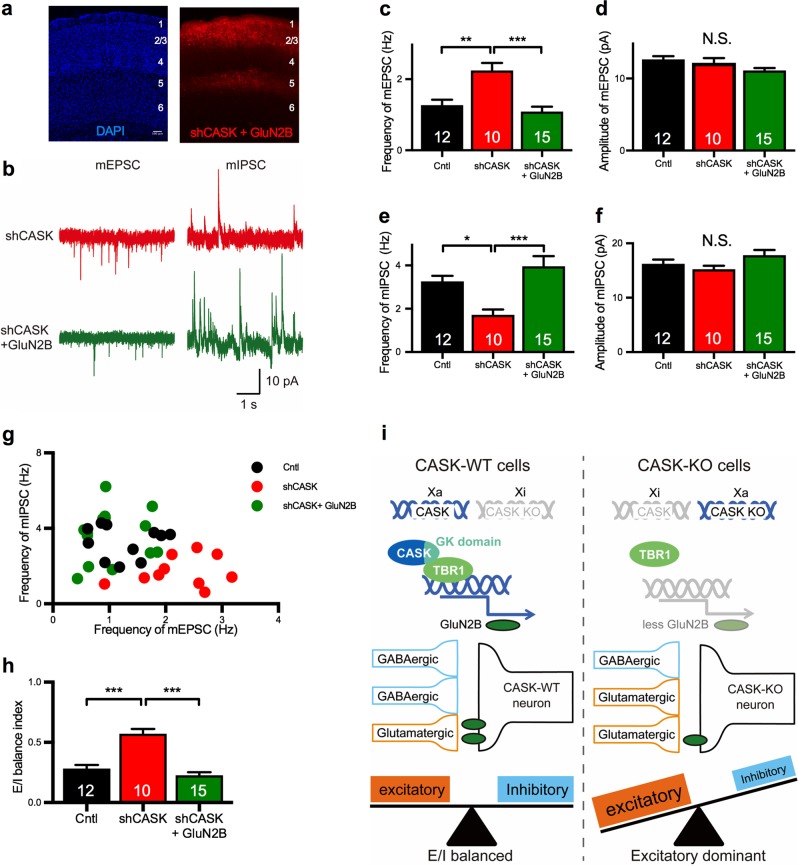
Co-transfection with GluN2B rescues the disrupted E/I balance in the spontaneous synaptic transmission in CASK-KD neurons. **a** Histological images of shCASK + GluN2B-transfected somatosensory cortex. The laminar structure was visualized by DAPI staining (left). tdTomato-labeled shCASK + GluN2B-transfected neurons were in layer 2/3 of the somatosensory cortex (right). Cortical layers are indicated by numbers. Scale bar: 100 µm. **b** Representative traces of the mEPSC (left) and mIPSC (right) from shCASK neurons (top) and shCASK + GluN2B neurons (bottom). Scale bars represent 10  pA (vertical axis) and 1 s (horizontal axis). **c**, **d** Frequency (**c**) and amplitude (**d**) of the mEPSC in control (black), shCASK (red), and shCASK + GluN2B (green) neurons. GluN2B transfection rescued the increased mEPSC frequency in shCASK-transfected neurons (animal numbers; Cntl *n* = 3, shCASK *n* = 3, shCASK + GluN2B *n* = 3). **e**, **f** Frequency (**e**) and amplitude (**f**) of the mIPSCs in control, shCASK, and shCASK + GluN2B neurons. GluN2B transfection rescued the decreased mIPSC frequency in the shCASK-transfected neurons. **g**. Distribution of the frequency of mEPSCs versus mIPSCs shown in a scatter plot. Each dot represents a single cell. **h** E/I balance index of neurons with each KD. **i** Molecular mechanism underlying the disruption of the E/I synaptic balance in CASK heterozygous female mice. Either the CASK-KO or WT allele is randomly inhibited (Xi) by XCI, and the activated X chromosome (Xa) determines the genotype of the cell. In CASK-KO neurons, GluN2B expression is down-regulated, resulting in a shifting of the E/I synaptic balance toward excitatory dominance. Statistical significance was determined by ANOVA and Bonferroni’s post hoc test (**c**–**f** and **h**). *p < 0.05, **p < 0.01, ***p < 0.001. Numbers on bars are the number of cells analyzed. N.S. not significant

To examine whether the overexpression of GluN2B rescued CASK-KD’s synaptic effects, we introduced a GluN2B construct together with shCASK into the pyramidal neurons in layer 2/3 of the somatosensory cortex by in utero electroporation, and analyzed the mPSCs. Overexpressing GluN2B in the CASK-KD neurons did not affect the brain morphology (Fig. 5a). Notably, the increased frequency of mEPSCs and decreased frequency of mIPSCs in the CASK-KD neurons were rescued to control levels by co-transfecting the GluN2B construct (Fig. 5b–f). The E/I balance expressed by scatter plot (Fig. 5g) and the E/I balance index were also restored to the control levels (Fig. 5h). These results indicated that the disruption of the E/I balance caused by CASK deficiency was due to the down-regulation of GluN2B function.

## Discussion

In the present study, we obtained the following findings: (1) CASK was subjected to XCI in mice, in which CASK-intact and -deficient neurons were distributed almost equally in a mosaic pattern in the brain. (2) The frequency of mEPSCs was increased and that of mIPSCs was decreased in both CASK^+/−^-KO and CASK-KD neurons through a developmental mechanism. (3) These effects were not rescued by overexpressing GK domain-mutant forms of CASK, which affect its interaction with TBR1. (4) The level of GluN2B mRNA was down-regulated in CASK-KD neurons, and overexpressing GluN2B in CASK-KD neurons rescued the E/I balance impairment (Fig. 5i).

Approximately 97% of the genes in mice and 85% of those in humans are predicted to be subject to XCI [47]. We demonstrated that CASK was subjected to XCI, by single-cell genotyping combined with patch-clamp electrophysiology. To our knowledge, this is the first study to use an XCI mechanism to analyze molecular functions in the brain by a single-cell genotyping technique. We observed an approximately 50–50% ratio between CASK^+/−^-WT and CASK^+/−^-KO neurons by random sampling of the pyramidal neurons in layer 2/3 of the somatosensory cortex in heterozygous CASK-KO mice (Fig. 1g). It is conceivable that the distribution of two different genotyped cells is skewed owing to biases in the timing or microenvironment upon XCI. While we focused on a small region of the brain in this study, overviewing the macroscopic distribution of CASK-deficient neurons in individual mice in relation to phenotype severity may help to elucidate the pathophysiology in human cases [20, 48].

Atasoy et al. [30] reported that the E/I balance is disrupted in CASK-KO cultured neurons. Both CASK^+/−^-KO and CASK-KD replicated this effect in the pyramidal neurons in acute brain slices. This effect appeared to be attributable to a developmental function of CASK, because conditional removal of CASK in the adult brain did not reproduce this phenotype (Fig. 2j, k). Given that differences in the frequencies of mPSCs were observed between CASK^+/−^-WT and CASK^+/−^-KO, but not between WT and CASK^+/−^-WT neurons, these phenotypes were related to the postsynaptic CASK deficiency. This scenario was supported by experiments performed in mosaic CASK-KD brain slices generated by in utero electroporation. While the synaptic effects of CASK have been studied primarily with respect to its presynaptic function [6, 7, 49, 50], our present study adds new insight that postsynaptic CASK influences the E/I balance of synaptic transmission. However, we cannot exclude the possibility that presynaptic CASK contributes to E/I function, and further studies are needed to clarify the presynaptic/postsynaptic effects of CASK on the maintenance of E/I synapse balance. As observed in cultured neurons, the membrane and firing properties in CASK^+/−^-KO or CASK-KD neurons in brain slices were unchanged. Thus, the seizures seen in patients with CASK mutations [19–21] are unlikely to be attributable to alterations in intrinsic excitability.

The distorted profiles of the mPSCs in CASK-KD neurons failed to be rescued by co-transfecting CASK lacking the GK domain. One well-documented function of CASK’s GK domain is to mediate target-gene transcription by interacting with TBR1 [13–15, 43, 44]. CASK’s GK domain binds to the C-terminal region of TBR1, and then the CASK/TBR1 complex is translocated to the nucleus, where it binds the T-element of target genes to induce transcription. Thus, we speculated that the observed synaptic phenotypes were mediated by a down-stream target gene of the CASK/TBR1 complex. This notion was further supported by our finding that the CASK-T704A mutant failed to rescue the synaptic phenotype caused by CASK deficiency (Fig. 3b, e).

We observed a reduction in the GluN2B mRNA level in CASK-KD neurons. CASK-KO and CASK-KD neurons also showed a reduced amplitude and shortened decay time constant of the NMDA receptor-mediated currents, suggesting that the GluN2B function was down-regulated. GluN2B expression is decreased in TBR1-knockout mice [15, 51–54]. Considering that CASK functions as a co-activator of TBR1, this decrease in GluN2B appeared to result from dysfunctional CASK/TBR1-mediated transcription. GluN2B-KD neurons showed an increased mEPSC frequency and decreased mIPSC frequency, mimicking the CASK-deficient synaptic phenotypes. Furthermore, overexpressing GluN2B restored the altered mPSCs in CASK-KD neurons.

The roles GluN2B in excitatory synaptic functions have been studied in GluN2B-KD and -KO hippocampal neurons, both of which exhibit an increased mEPSC frequency [46, 55–57], as we observed. In addition, we analyzed the effect of GluN2B deficiency on inhibitory synaptic functions and found that the change was shifted in the opposite direction. Changes in the frequency, but not amplitude, of mPSCs suggest that the anomaly is in the presynapses. The absence of a PPR phenotype in both E/I synaptic transmission indicated that the number of functional synapses projecting onto CASK-deficient neurons may be altered.

In summary, our study provides evidence for a molecular mechanism in which CASK deficiency downregulates the GluN2B subunit of NMDA receptors, due to the loss of CASK’s GK domain, resulting in disruption of the E/I synaptic balance in the brain. Further studies addressing how a mosaic deficiency of CASK in neural circuits causes defective brain functions are needed to understand the pathophysiology of CASK-deficient diseases.

## Supplementary information

Supplementary Figure Legends

Full Methods

Supplementary Figure 1

Supplementary Table 1

Supplementary Figure 2

Supplementary Figure 3

Supplementary Figure 4

Supplementary Figure 5

Supplementary Figure 6

Supplementary Figure 7

Supplementary Figure 8

Supplementary Figure 9

Supplementary Figure 10
