# Correction: Deficiency of calcium/calmodulin-dependent serine protein kinase disrupts the excitatory-inhibitory balance of synapses by downregulating GluN2B

**DOI:** 10.1038/s41380-019-0362-z

**Published:** 2019-01-31

**Authors:** Takuma Mori, Enas A. Kasem, Emi Suzuki-Kouyama, Xueshan Cao, Xue Li, Taiga Kurihara, Takeshi Uemura, Toru Yanagawa, Katsuhiko Tabuchi

**Affiliations:** 1 Department of Molecular and Cellular Physiology, Institute of Medicine, Academic Assembly, Shinshu University, 390-8621 Nagano, Japan; 2 Department of Zoology, Faculty of Science, Kafr Elsheikh University, 33511 Kafr Elsheihk, Egypt; 3 Institute for Biomedical Sciences, Interdisciplinary Cluster for Cutting Edge Research, Shinshu University, 390-8621 Nagano, Japan; 4 CREST, JST, 332-0012 Saitama, Japan; 5 Department of Oral and Maxillofacial Surgery, Faculty of Medicine, University of Tsukuba, 305-8575 Ibaraki, Japan; 6 PRESTO, JST, 332-0012 Saitama, Japan

**Keywords:** Physiology, Neuroscience

**Correction to:** **Molecular Psychiatry**;

10.1038/s41380-018-0338-4;

published online 4 January 2019

This article was originally published under standard licence, but has now been made available under a [CC BY 4.0] license. The PDF and HTML versions of the paper have been modified accordingly.

