# Supplementary material for: Deficiency of calcium/calmodulin-dependent serine protein kinase disrupts the excitatory-inhibitory balance of synapses by down-regulating GluN2B
Source: Mol Psychiatry. 2019 Jan 4;24(7):1079–92. doi: 10.1038/s41380-018-0338-4 (PMC6756202; doi:10.1038/s41380-018-0338-4)
Supplement: Supplementary file 1 — Supplementary Figure Legends [file 41380_2018_338_MOESM1_ESM.docx]

Supplemental Figure 1. Brain morphology and seizure threshold of CASK-deficient mice.

a. Brains of 5-week-old CASK+/+ (left) and CASK+/- (right) mice. Hypotrophy of the cerebellum was seen in the CASK+/- brain (arrow). Scale bar: 1 cm.

b. Thickness of the cortical layers in WT and CASK mutant mice. CASK+/- female (*n* = 4 animals, red), CASKY/+ male (*n* = 4 animals, grey), CASK+/+ female (*n* = 4 animals, black). No statistically significant difference was detected by ANOVA or Bonferroni's post-hoc test.

c. Time course of PTZ-induced seizures observed in CASK+/- (n = 7 animals, grey) and CASK +/+ (n = 6, black) females. Seizure was scored based on the Racine’s method. Statistial significant was tested with Mann-Whitney rank-sum test ( *p < 0.05, **p < 0.01, ***p < 0.001)

d. Highest seizure scores during ten minutes after PTZ injection to CASK+/- and CASK+/+ females. Score is higher in CASK+/- than CASK +/+ (p < 0.001, Mann-Whitney rank-sum test).

Supplementary Figure 2. Imbalance of excitatory and inhibitory synaptic inputs in hippocampal CA1 pyramidal neurons.

a. Representative traces of the mEPSC (top) and mIPSC (bottom) recorded in CASK-expressing (CASK+/- WT, left) and CASK-deficient (CASK+/- -KO, right) neurons.

b. Graphs of the frequency and amplitude of mEPSCs in hippocampal CA1 pyramidal neurons. The frequency of the mEPSC was increased in CASK+/- -KO neurons.

c. Graphs of the frequency and amplitude of mIPSCs. The frequency of the mIPSC in CASK+/- -KO neurons was decreased. Statistical significance was examined by Student’s *t-*test (p > 0.1). (CASK^+/-^ female n=5)

Supplementary Figure 3. CASK-KD efficiency, and localization of CASK-KD neurons.

a. Knock-down efficiency of shCASK was examined in single neurons. The mRNA level of CASK was decreased to 13.4% of the control level.

b. Histological images of the shRNA-CASK-transfected somatosensory cortex. The laminar structure was visualized by DAPI staining (left). The tdTomato-labeled shCASK neurons were in layer 2/3 of the somatosensory cortex (right). Cortical layers are indicated by numbers. Scale bar: 100 μm.

Supplementary Figure 4. Dendritic spine density was unchanged by CASK knockdown.

a. Representative images of the dendrites of cortical pyramidal neurons transfected with control (top) and CASK-knockdown (bottom) vectors. Scale bar = 2 nm.

b. Graph of dendritic spine density. Dendritic spine density was unchanged by CASK knockdown. Numbers on bars indicate the number of dendrites analyzed. Statistical significance was examined by Student’s t test (p > 0.1). (Cntl mice n=3, shCASK mice n=3)

Supplementary Figure 5. Membrane properties and excitability of CASK-knockdown cortical neurons.

a-e. Resting membrane potential (a), action potential (AP) threshold (b), AP amplitude (c), AP half-time width (d), and input resistance (e) of neurons under four different KD conditions.

f. Input (injected currents) and output (spike number) relationship under four different KD conditions. Sample number: 11 neurons from three animals for each condition. No statistically significant difference was detected by ANOVA or Bonferroni's post-hoc test.

Supplementary Figure 6. Release probability was not altered by CASK-KD.

a. Sample traces of AMPA receptor-mediated evoked synaptic currents (AMPA-eEPSCs) in control, shRNA-CASK, and shRNA-CASK + rescue construct transfected neurons. Paired responses with different intervals were superimposed in each trace. Scale bars represent 50 pA (vertical axis) and 0.2 s (horizontal axis).

b. Paired pulse ratio of AMPA-eEPSCs in control, shCASK, and shCASK + rescue construct transfected neurons.

c. Sample traces of GABAA receptor-mediated evoked synaptic currents (eIPSCs) in control, shCASK, and shCASK + rescue construct transfected neurons. Paired responses with different intervals were superimposed in each trace. Scale bars represent 200 pA (vertical axis) and 0.2 s (horizontal axis).

d. Paired pulse ratio of eIPSCs in control, shCASK and shCASK + rescue construct transfected neurons.

Supplementary Figure 7. Mouse CASK deletion mutants used for rescue experiments.

a. Schematic of CASK deletion mutants. Full-length CASK (FL) consists of 5 subdomains: a Calmodulin dependent serine threonine kinase domain (CAM/STK), Lin2/Lin7 domain (LIN), PDZ domain (PDZ), Src Homology 3 domain (SH3), and Guanylate kinase domain (GK). The CASKTA mutant (TA) was generated by substituting alanine for threonine at position 704 (T704) (arrow). The mouse CASK T704 in this study corresponds to T724 in the rat CASK. The HA epitope tag was added to the N-terminus of each CASK mutant.

b. Western blotting analysis of the *in vitro* expression of CASK deletion mutants. Lysates from HEK293T cells transfected with expression vectors encoding one of the HA-tagged CASK variants were loaded in each lane. CASK proteins were detected using an anti-HA antibody. Lane M, molecular weight standards.

c. Images for the immunohistochemical detection of CASK deletion mutants. HA immunoreactivity (green) was examined in 50-μm brain sections. tdTomato signals are shown in red and DAPI in blue. Scale bar = 100 μm.

Supplementary Figure 8. Expression of TBR1 in cortical layer 2/3 neurons.

a. Cortical laminar structure visualized by DAPI staining. Numbers indicate cortical layers. White dashed line indicates the border between layers 1 and 2/3.

b. Immunohistochemical staining of TBR1.

c. Merged image of A and B. Scale bar: 100 μm.

Supplementary Figure 9. Efficiency of GluN2B-KD, and localization of GluN2B-KD neurons.

a. KD efficiency of shGluN2B in cortical neurons.

b. Histological images of the shGluN2B-transfected somatosensory cortex. The laminar structure was visualized by DAPI staining (left). The tdTomato-labeled shGluN2B-transfected neurons were in layer 2/3 of the somatosensory cortex (right). Cortical layers are indicated by numbers. Scale bar: 100 μm.

Supplementary Figure 10. Paired pulse ratio of excitatory and inhibitory synaptic inputs in GluN2B KD neurons.

a. Sample traces of AMPA-mediated evoked synaptic inputs. Two serial responses were evoked with different intervals. Scale bars represent 50 pA (vertical axis) and 0.2 s (horizontal axis).

b. Paired pulse ratio of evoked AMPA inputs with different intervals (n = 20 control neurons from four animals; n = 14 shGluN2B-transfected neurons from four animals).

c. Sample traces of GABAA-mediated evoked synaptic inputs. Scale bars represent 200 pA (vertical axis) and 0.2 s (horizontal axis). d. Paired pulse ratio of evoked GABAA inputs with different intervals (n = 11 control neurons from three animals; n = 9 shGluN2B transfected neurons from three animals). No statistically significant difference was detected by an unpaired t-test.
