# Supplementary material for: Deficiency of calcium/calmodulin-dependent serine protein kinase disrupts the excitatory-inhibitory balance of synapses by down-regulating GluN2B
Source: Mol Psychiatry. 2019 Jan 4;24(7):1079–92. doi: 10.1038/s41380-018-0338-4 (PMC6756202; doi:10.1038/s41380-018-0338-4)
Supplement: Supplementary file 2 — Full Methods [file 41380_2018_338_MOESM2_ESM.docx]

**Methods**

All animal procedures were approved by the Animal Care and the Use Committee of Shinshu University School of Medicine.

**Animals.** CASK knockout mice were obtained by crossing female mice carrying CASK-floxed (B6;129-Cask^tm1Sud^/J, JAX Stock #006382) and ZP3-Cre (C57BL/6-Tg(Zp3-cre)93Knw/J, JAX Stock #003651) with C57BL/6J male mice. Genotyping of CASK KO mice were done by PCR using primers described in Supplementary Table 1.

**Construction of plasmids.** shRNA constructs: target sequences for mouse CASK 5'-ATCCATGAGCAGGGGCTGA-3', mouse GluN2B; 5'-GGATGAGTCCTCCATGTTCTT-3'[^41^](#_ENREF_41) and scrambled; 5'-TTCTCCGAACGTGTCACGT-3' were cloned into L309 backbone vector[^60^](#_ENREF_60).

pCAGGS-CASK: mouse full-length CASK cDNA, obtained from mouse brain tissues by RT-PCR, was cloned into pTOPO vector and introduced synonymous mutations in the shRNA target sequence (5'-ATTCATGAACAGGGCCTGA-3'; mutation sites are underlined) located in the guanylate kinase domain coding region, followed by re-subcloning into pCAGGS vector. For constructing CASK deletion mutant expression vectors (CASKΔCAM, CASKΔLIN, CASKΔPDZ, CASKΔSH3, and CASKΔGK), PCRs were performed on pTOPO-mouse CASK using chimeric primers lacking each domain coding region with 15-20 bp overlapping at 3’ ends. CASK^TA^ was generated by site-directed mutagenesis using primers containing T704A mutation in the middle. forward; 5'-CTGCCAGCATTCAAAAGGAAAGCATTAGTCTTATTAGGTGCAC-3', and rev; 5'-GTGCACCTAATAAGACTAATGCTTTCCTTTTGAATGCTGGCAG-3'. To confirm expression levels of mutant CASKs, duplicated (2x) HA epitope sequence was added to the N terminals.

pCAGGS-TVA-tdTomato: TVA-tdTomato fusion protein was constructed by TVA950 fused by tdTomato and the coding sequence was cloned into pCAGGS vector.

pEB6-GluN2B was constructed by insertion of mouse GluN2B cDNA to pEB6-MCS.

**In utero electroporation.** *In utero* electroporation was performed as essentially described previously [^31^](#_ENREF_31)^,^[^40^](#_ENREF_40). Briefly, pregnant ICR mice at E15.5 were anesthetized, and the uterine horns were exposed. Approximately 1 μl of DNA solutions containing 0.01% fast green were injected into the lateral ventricles of embryos using a pulled borosilicate glass capillaries (B120F-4; World Precision Instruments). The DNA solutions contained 1.0 μg/ul knockdown vector, 2.0 μg/ul of a rescue vector (if applicable) and 0.7 μg/ul of the tdTomato expression vector. The concentrations of plasmids were determined to ensure that almost all the tdTomato positive cells were co-transfected with both rescue and knock-down vectors. The head of embryo in the uterus was placed between tweezers-type electrodes with 5 mm diameter (CUY650P5; NEPA Gene). The embryos were subjected to five square electric pulses (35 V, 50 msec, 1 Hz) using electroporator (CUY21E; NEPA Gene). After electroporation, the embryos were returned to the abdominal cavity to allow continuous development. Transfected pups were identified at P0-2 by tdTomato signals through scalp using an LED handy light with a RFP filter. The brains with abnormal morphology were excluded from the experiments.

**Electrophysiology.** P14-P18 mouse brains were removed and placed immediately in ice-cold slicing artificial corticospinal fluid (ACSF, in mM: 85 NaCl, 75 sucrose, 2.5 KCl, 1.25 NaH_2_PO_4_, 24 NaHCO_3_, 25 glucose, 0.5 CaCl_2_, and 4 MgCl_2_) saturated with 95% O_2_/5% CO_2_ for 2 min. The chilled brains were trimmed coronally with razor blades and placed in a vibratome chamber (Campden 7000smz). Three hundred fifty-μm-thick coronal sections were transferred to a recovery chamber filled with recording ACSF (in mM: 126 NaCl, 2.5 KCl, 1.25 NaH_2_PO_4_, 26 NaHCO_3_, 10 glucose, 2 CaCl_2_, and 2 MgCl_2_), followed by incubated at 32°C for 30 min, and then at room temperature for 1 hour. In current clamp experiments, pyramidal neurons were patched with glass pipettes (4-8 M ohm) filled with a potassium based intra-cellular solution (ICS, in mM: 130 K Gluconate, 6 KCl, 10 HEPES, 1 EGTA, 2.5 MgCl_2_, 2 magnesium ATP, 0.5 sodium GTP, 10 phosphocreatine sodium, 290 mOsm) under a fluorescence microscopy. Resting membrane potential was measured immediately after establishing whole cell recording. Hyperpolarizing and depolarizing step pulses (700 ms) were applied to characterize neuronal firing property. Membrane potential at which the temporal rate of the potential reached to 10 mV/ms was defined as action potential threshold. Postsynaptic responses were measured in voltage clump mode using cesium based ICS (in mM: 130 CsOH, 130 Gluconic acid, 6 CsCl, 10 HEPES, 1 EGTA, 2.5 MgCl_2_, 2 magnesium ATP, 0.5 sodium GTP, 10 phosphocreatine sodium, 290 mOsm). Miniature postsynaptic currents were recorded in the presence of 1 uM tetrodotoxin (Abcam). Membrane potential was held at -60 mV for mEPSCs and 0 mV for mIPSCs. Evoked postsynaptic currents were triggered with 0.1 msec current injections by a nichrome-wire electrode placed at position 100-150 um from the soma of neurons recorded. For evoked AMPA-EPSC, 100 μM picrotoxin was added in bath solution with holding potential at -60 mV. For GABA-IPSC, 10 μM of DNQX was added in bath solution with holding potential at 0 mV. For NMDA-EPSC, 100 μM picrotoxin was added in bath solution with holding potential at 40 mV. Paired-pulse responses were recorded at inter-stimulus intervals ranging from 30 ms to 200 ms. Pired-pulse ratio was calculated by dividing the amplitude of second response by that of first one. To calculate the NMDA to AMPA ratio, the amplitude of NMDA current at 50 ms after the onset was divided by peak amplitude of AMPA current. All data were acquired at 10kHz with EPC10 double amplifier (HEKA) operated by Patch Master software (HEKA). Access resistance was monitored throughout the recording, and cells with access resistance over 25 MΩ or changed over 25% during the recording were rejected. Data analysis was performed with Mini Analysis Program (Synaptosoft) and Igor Pro (WaveMetrics). E/I balance index was calculated by averaging (Freq^mEPSC^)/(Freq^mEPSC^+Freq^mIPSC^), where Freq^mEPSC^ and Freq^mIPSC^ represent the frequency of mEPSC and mIPSC, respectively.

**AAV injection.** All AAV vectors were purchased from the Vector Core of University of North Carolina. CASK-floxed mice were deeply anesthetized by isoflurane and placed on stereotactic device (Narishige). Approximately 300 nl of AAV2/5-hSyn-GFP-Cre or AAV2/5-hSyn-GFP were injected into the somatosensory cortex of the mice at postnatal 10-13 days and returned to their mother. The mice were survived for two weeks and make acute brain slices (described above).

**Single Cell RT-PCR.** Single cell RT-PCR was performed as described previously with modifications. Cytoplasm of recorded neuron was aspirated through patch pipettes and transferred into a 0.2-ml PCR tube containing 2× RT master mix (High Capacity cDNA Reverse Transcription Kits; Applied Biosystems). cDNA was synthesized according to the manufacturer's instruction. Single cell cDNA was amplified by two round semi-nested PCR with primers listed on Supplementary Table 1: the first PCR, one cycle of 98°C for 2 min; 20 cycles of 94°C for 10 sec, 60°C for 30 sec and 68°C for 30 sec; the second nested PCR, one cycle of 98°C for 2 min; 40 cycles of 94°C for 10 sec, 60°C for 30 sec and 68°C for 30 sec. All PCR was performed using KOD FX Neo (TOYOBO). PCR products were run in 2% of agarose gel. The animals were genotyped before recording and recorded cells were genotyped after the recording for blind experiments.

**Cell culture and western blotting.** HEK-293T cells (0.5 X 10^6^) were plated on 6-well plates on the day before the transfection. 2 μg of pCAGGS-tdTomato and 2 μg of expression vector of each CASK mutant were transfected with polyethylenimine. Forty-eight hours after transfection, cells were collected and lysed in lysate buffer (1mM EDTA, 100 mM NaCl, 1% Triton X-100, in 20mM HEPES pH 7.4) mixed with protease inhibitor cocktail (Nacalai, JAPAN). Cell lysates were added to 2x SDS sample buffer, boiled for 5 min, and loaded on Polyacrylamide gel (7.5 %) and transferred on PVDF membrane. Transferred membrane was incubated with primary antibody against HA epitope tag (1:1000, Covance) at 4 °C overnight. After washing, membrane was incubated with secondary antibody (1:20000, IRdye 800CW) at room temperature for 30 min, and washed. The signals were detected and analyzed on Odyssey imaging system (LI-COR Biotechnology).

**Morphological analysis.** Under deep anesthesia, mice were perfused transcardially with ice-cold phosphate-buffered saline (PBS, pH 7.4), followed by 4% Paraformaldehyde in PBS. Fifty-μm-thick coronal sections were prepared with microslicer (VT1200S; Leica Biosystems) or sliding microtome (REM-700, Yamato Kokhi Industrial). The sections were washed with PBS, blocked with PBS containing 1% bovine serum albumin, 0.1% Triton-X100 and 10% of normal donkey serum and incubated with rabbit anti-TBR1 antibody (1:200, Abcam), mouse anti-CASK antibody (1:400, NeuroMAB), or mouse anti-Hemagglutinin (HA) antibody (1:200, Covance). After overnight incubation with primary antibodies, the brain sections were washed with PBS containing 0.1% Triton-X100 and incubated with Alexa 488-conjugated donkey antibody against rabbit IgG or Cy3-conjugated donkey antibody against mouse IgG (Jackson immunoresearch), respectively, for 2-3 hours at room temperature. After further washing brain sections with PBS, brain sections were mounted on a slide glass, counterstained with DAPI, and coverslipped. Fluorescence images were taken with a confocal laser-scanning microscope (TCS SP8; Leica Microsystems).

**Measurement of mRNA and protein levels in knock-down culture neurons.**

RT-qPCR was performed on RNA isolated from primary cortical neuronal culture infected with lentivirus. Lentivirus was produced by transfection of lentiviral plasmid (L309) containing shRNA module and helper plasmids (VSVG, pMDLg/pRRE, and pRSV-REV) to HEK293T cells using polyethylene imine. Viral particles were concentrated using Lenti-X Concentrator (Clontech). Primary cortical neuron cultures were prepared from neonatal ICR mice at postnatal day 0. The cerebral cortex was treated with 1% trypsin and 0.1% DNaseI (SIGMA) for 5 min at room temperature, followed by washing with Neurobasal-A (Invitrogen) containing 5% fetal bovine serum (FBS). The cells were dissociated by passing through fire-polished Pasteur pipette repeatedly in phosphate-buffered saline containing 0.01% DNase I, 0.03% Trypsin inhibitor (SIGMA), and 2 mM MgCl_2_. The cells were plated at a density of 1 × 10^5^ cells/cm^2^ on the poly-L-lysine (SIGMA) and mouse laminin (Invitrogen) coated-cell culture dish. The cells were cultured in Neurobasal-A containing 2% B-27 supplement (Invitrogen), 1% Glutamax (Invitrogen), 1% Pen/Strep (Invitrogen), and 5% FBS for the first day after plating. The medium was replaced on the next day with the one excluding FBS. Lentivirus was applied to the primary cortical neurons on 3-4 days *in vitro* and cells were collected on 14 days *in vitro*. Collected cells were lysed and total RNA was extracted with Trizol reagent (Invitrogen). cDNA was synthesized using High Capacity cDNA Reverse Transcription Kit. 300 ng of cDNA and primers (listed on supplementary table 1) was added to KOD SYBR qPCR Mix (Toyobo). Real time PCR reactions were run and analyzed on StepOnePlus (Thermo Fisher) with GAPDH as an internal control. For immunoblot analysis, we used rabbit anti-GluN2A and GluN2B antibodies (GluRe1C-Rb-Af542 and GluRe2C-Rb-Af300, Frontier Institute) and mouse anti-beta-actin antibody (Medical and Biomedical Laboratories) as primary antibodies and IRDye-labeled goat anti rabbit or mouse IgG as secondary antibodies. Neurons were lysed with 4x sample buffer and the immunoblots were performed as described above.

**Pentylenetetrazol induced seizure.**

Female mice (7 to 8-week old) were injected pentylenetetrazol (PTZ) in saline (50mg/kg, i.p.). Development of PTZ induced seizure was scored based on a modified Racine's scale: (0) No abnormal movements. (1) Reduced motility and prostate position. (2) Partial clonus. (3) Generalized clonus including extremities. (4) Tonic-clonic seizure with rigid paw extension. (5) Death. Score was determined every minute during 10-min observation.

**Sample size and Statistical analysis.** Samples sizes were determined based on established practice and on our previous experience in respective assays^29,34,36^. The number of independent samples (e.g. neurons) is indicated on the graphs and the numbers of animals indicated in the figure legends. All values represent the average of independent experiments ± SEM. The variance among analyzed samples was similar. Statistical significance was determined by Student's t-test (for two groups) or one-way ANOVA followed by Bonferroni's post-hoc test (for multiple groups). Statistical analysis was performed with Prism 6.0 (Graphpad Software Inc.). Statistical significance is indicated by asterisks (* p < 0.05, ** p < 0.01, *** p < 0.001).
