# Supplementary figures and images for: Deficiency of calcium/calmodulin-dependent serine protein kinase disrupts the excitatory-inhibitory balance of synapses by down-regulating GluN2B
Source: Mol Psychiatry. 2019 Jan 4;24(7):1079–92. doi: 10.1038/s41380-018-0338-4 (PMC6756202; doi:10.1038/s41380-018-0338-4)

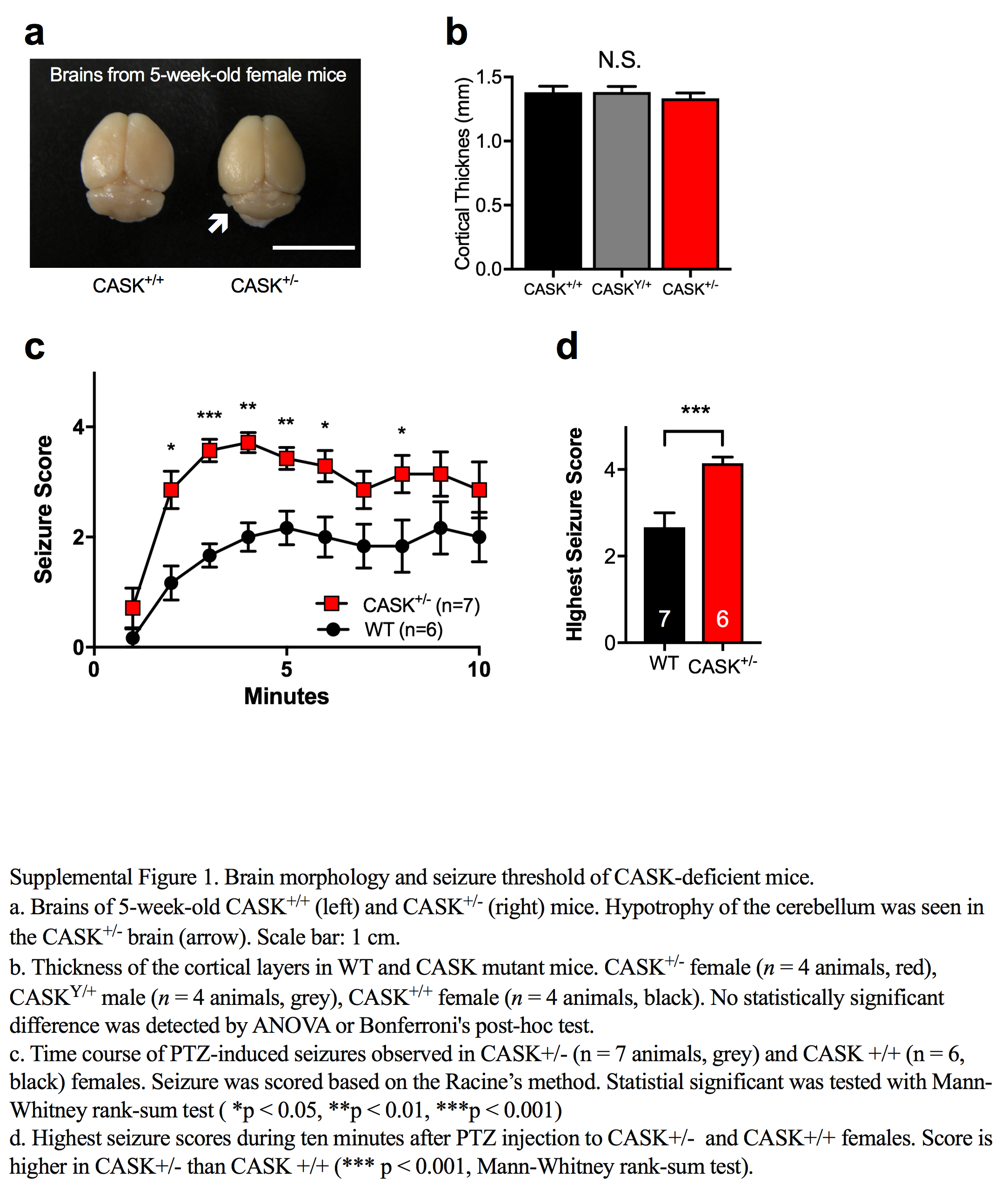

Supplement: Supplementary file 3 — Supplementary Figure 1 [file 41380_2018_338_MOESM3_ESM.tif]

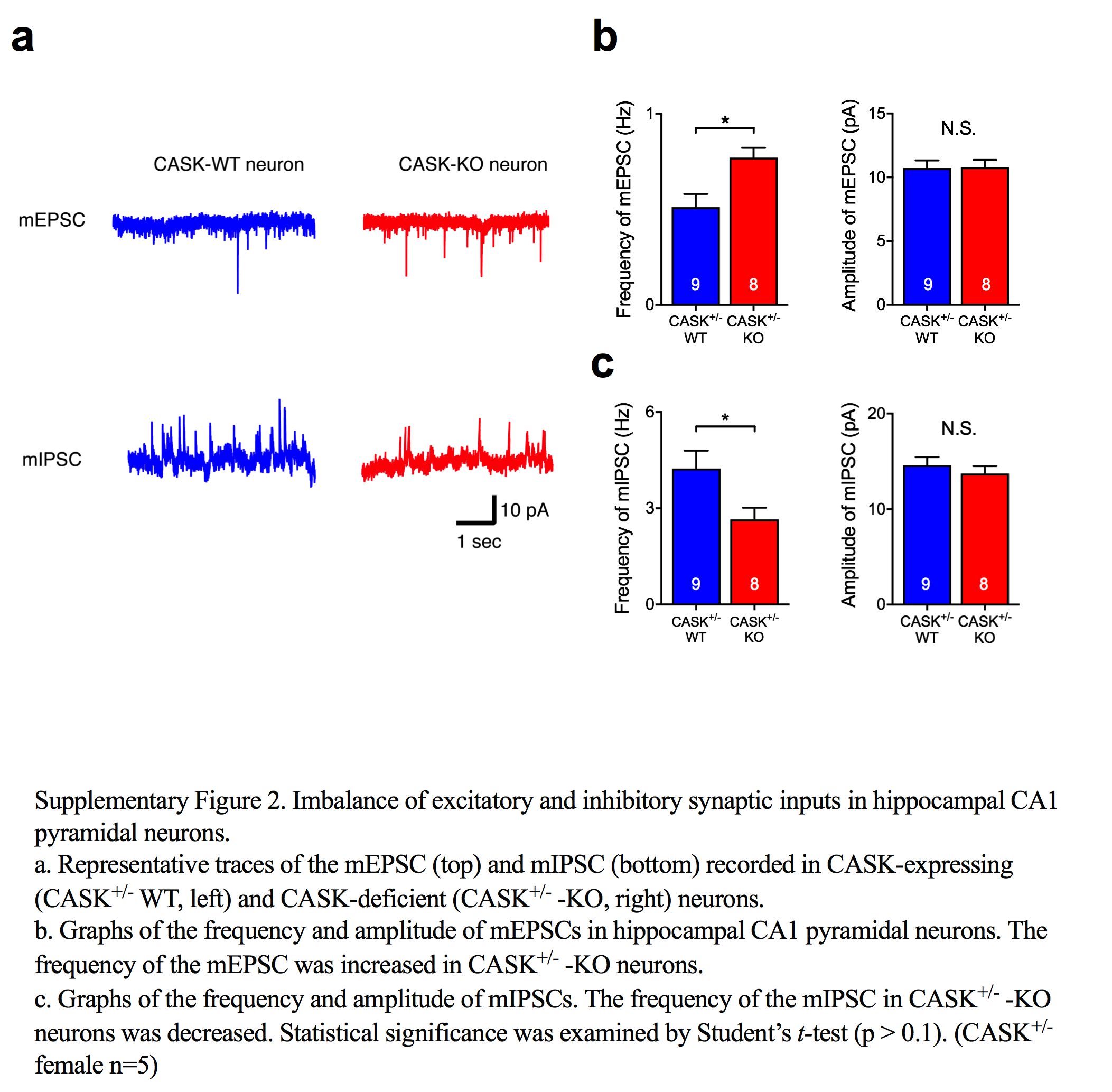

Supplement: Supplementary file 5 — Supplementary Figure 2 [file 41380_2018_338_MOESM5_ESM.tif]

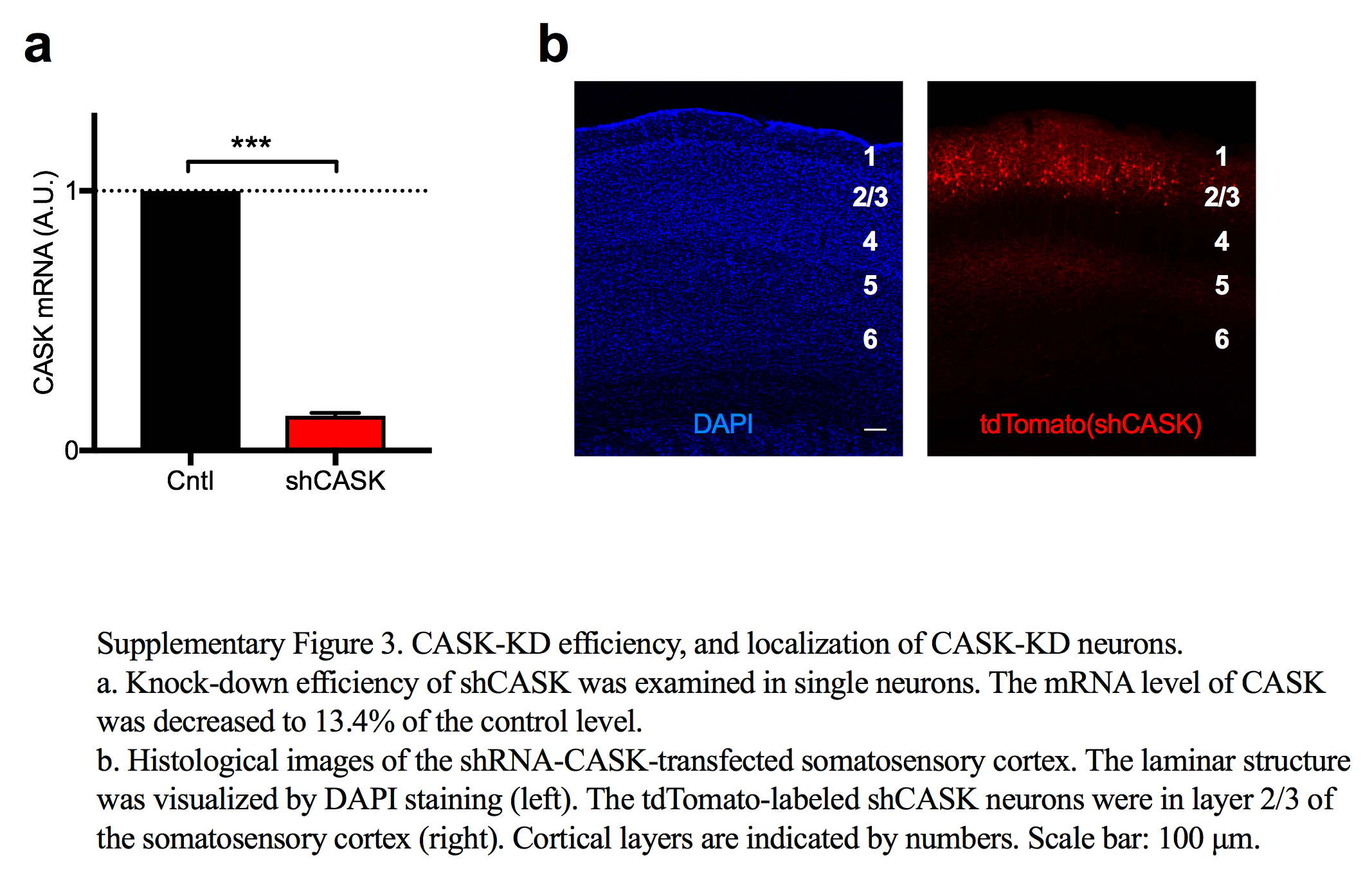

Supplement: Supplementary file 6 — Supplementary Figure 3 [file 41380_2018_338_MOESM6_ESM.tif]

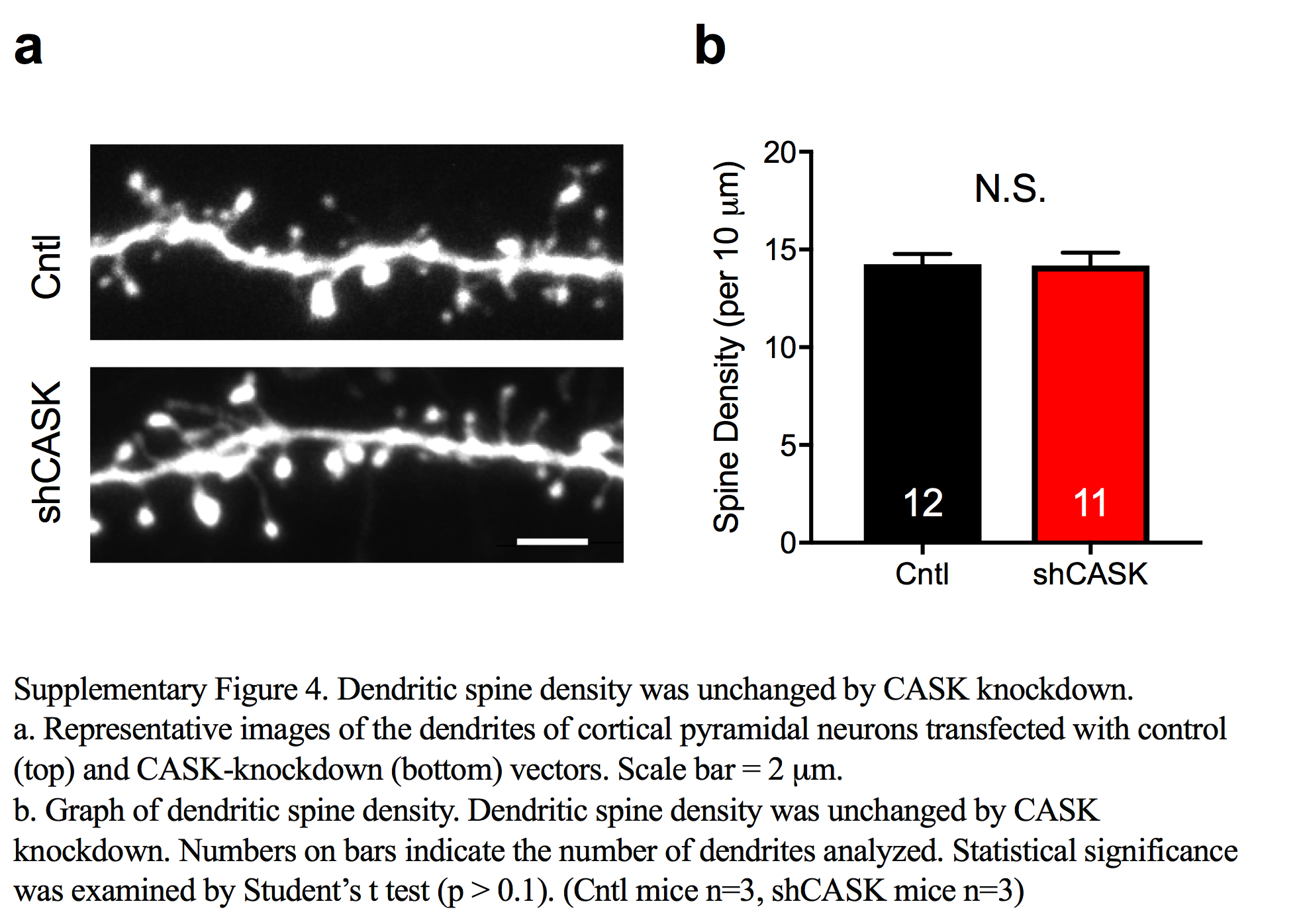

Supplement: Supplementary file 7 — Supplementary Figure 4 [file 41380_2018_338_MOESM7_ESM.tif]

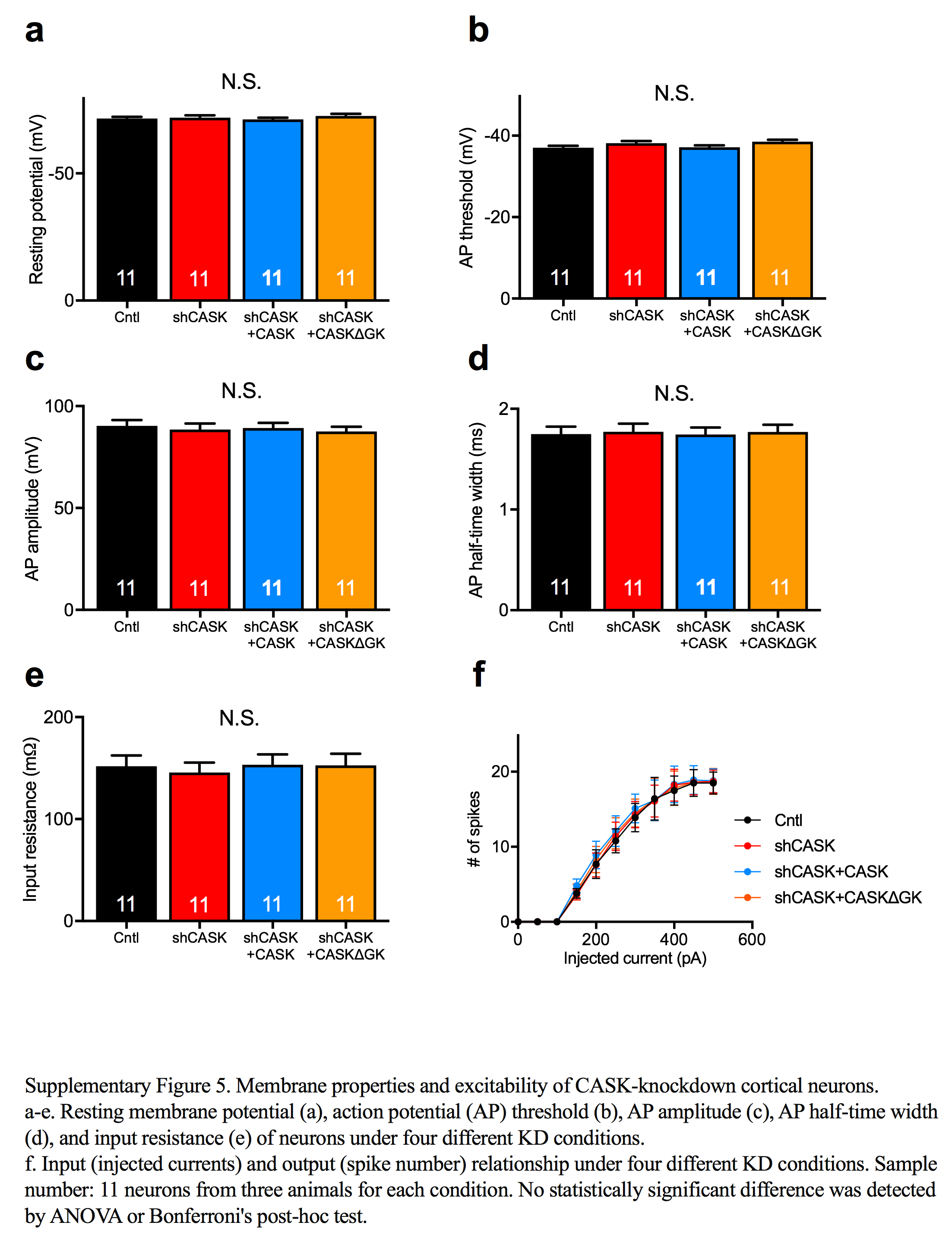

Supplement: Supplementary file 8 — Supplementary Figure 5 [file 41380_2018_338_MOESM8_ESM.tif]

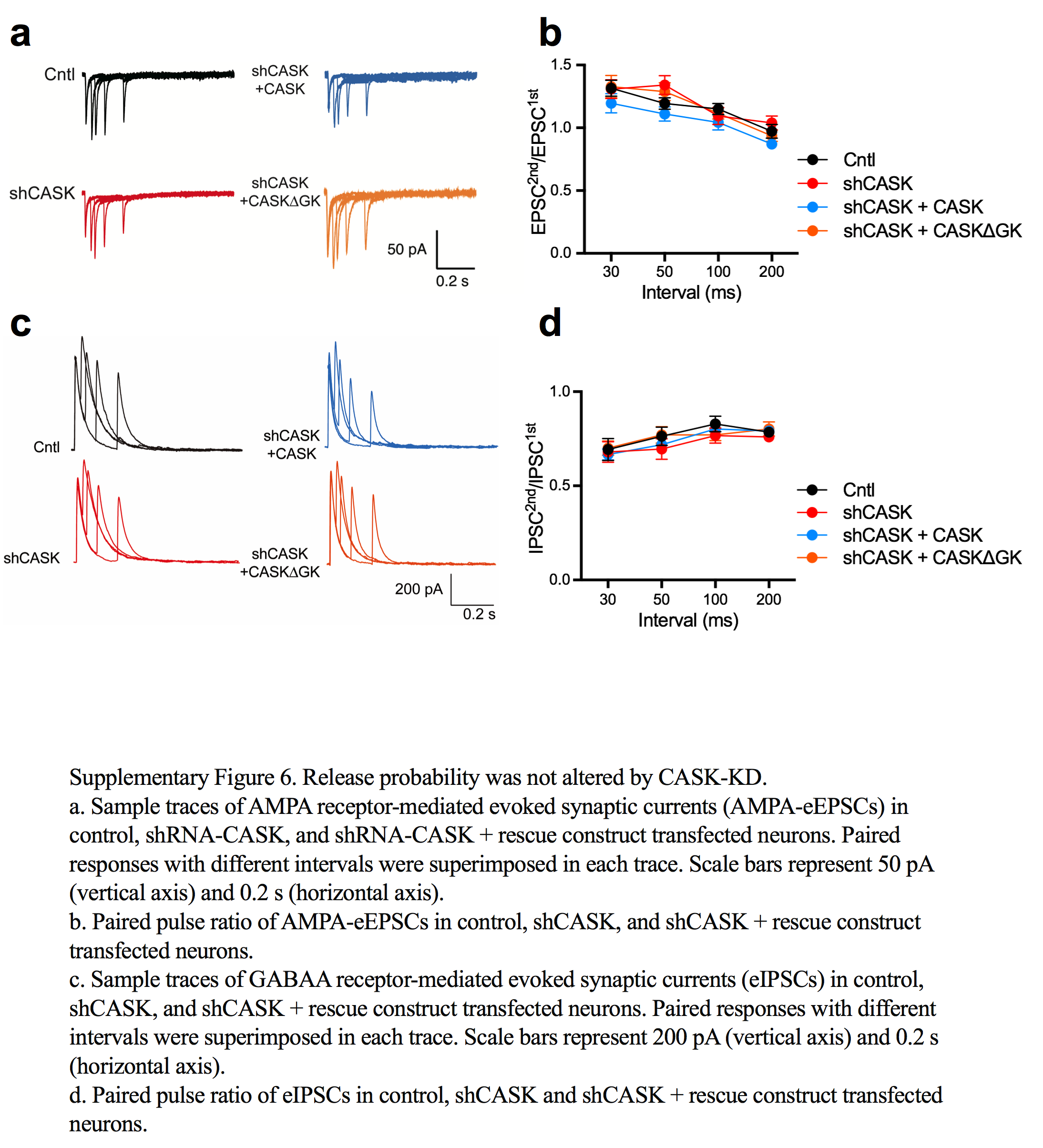

Supplement: Supplementary file 9 — Supplementary Figure 6 [file 41380_2018_338_MOESM9_ESM.tif]

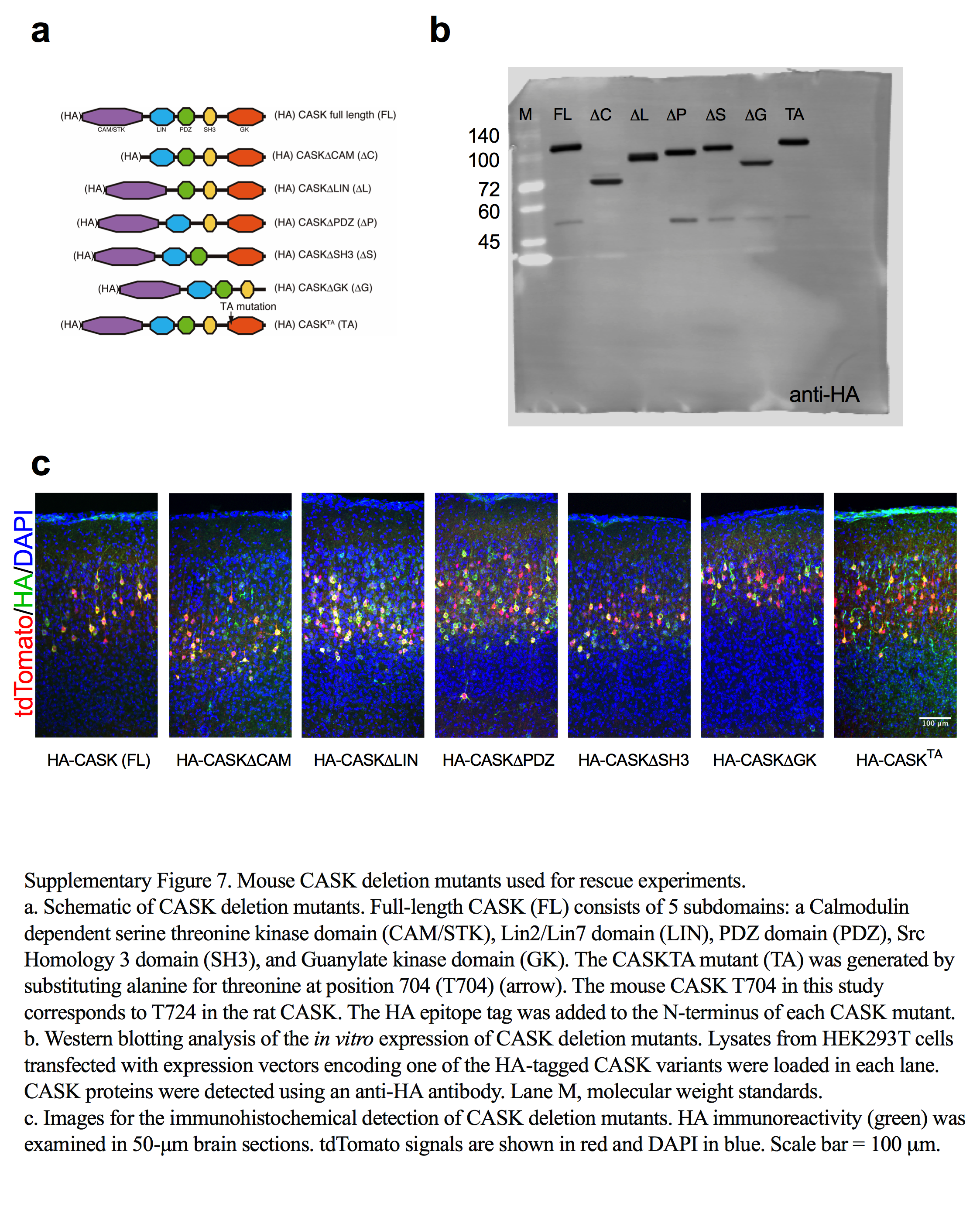

Supplement: Supplementary file 10 — Supplementary Figure 7 [file 41380_2018_338_MOESM10_ESM.tif]

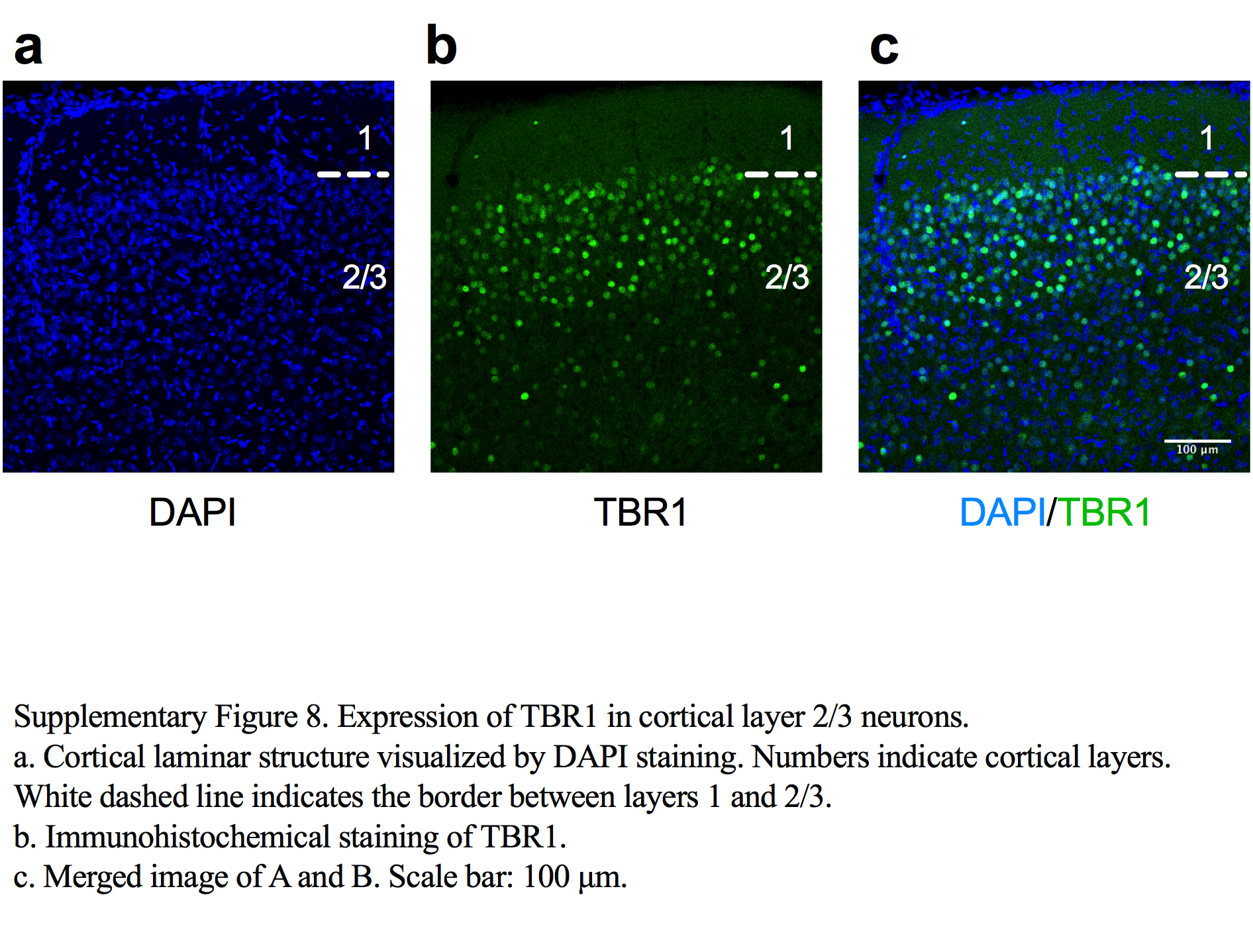

Supplement: Supplementary file 11 — Supplementary Figure 8 [file 41380_2018_338_MOESM11_ESM.tif]

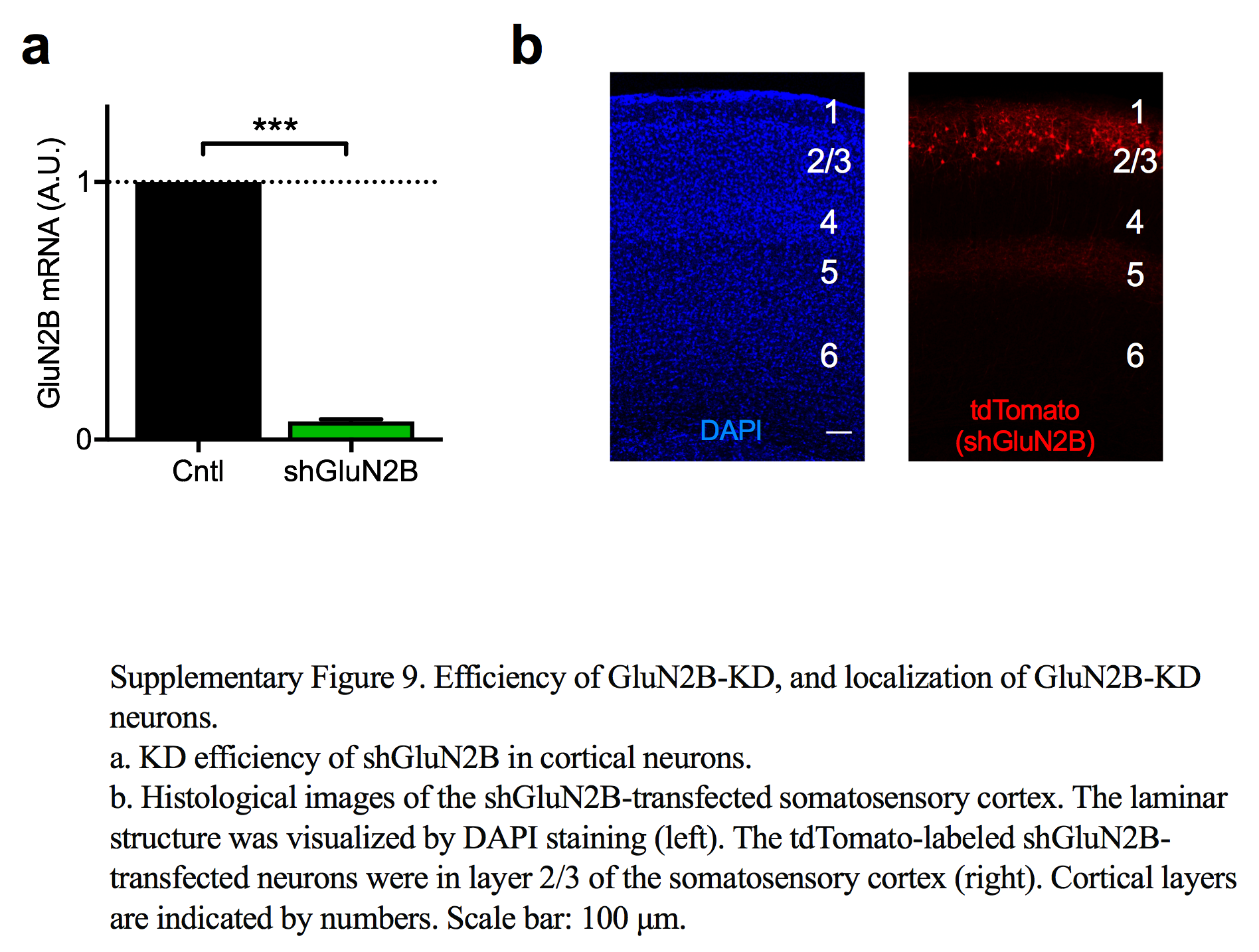

Supplement: Supplementary file 12 — Supplementary Figure 9 [file 41380_2018_338_MOESM12_ESM.tif]

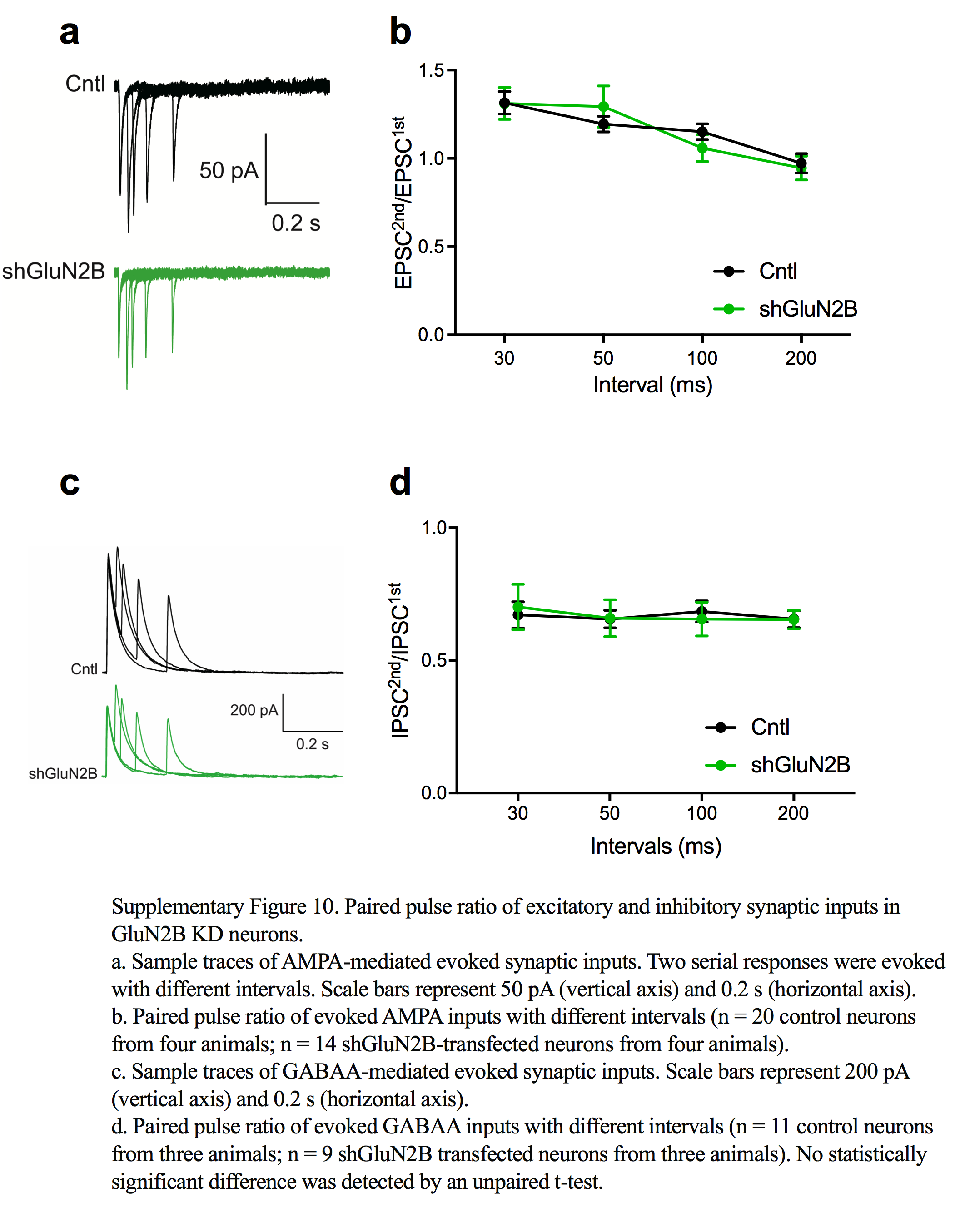

Supplement: Supplementary file 13 — Supplementary Figure 10 [file 41380_2018_338_MOESM13_ESM.tif]
