# Supplementary material for: Deficiency of calcium/calmodulin-dependent serine protein kinase disrupts the excitatory-inhibitory balance of synapses by down-regulating GluN2B
Source: Mol Psychiatry. 2019 Jan 4;24(7):1079–92. doi: 10.1038/s41380-018-0338-4 (PMC6756202; doi:10.1038/s41380-018-0338-4)
Supplement: Supplementary file 4 — Supplementary Table 1 [file 41380_2018_338_MOESM4_ESM.docx]

Supplemental Table 1

Primers used in this study

Genotyping Type Sequence 5' to 3'

ZP3-Cre Forward GAAGATCTTCCAATTTACTGACCGTACAC

Reverse CCATGAGTGAACGAACCTGGTCGA

CASK floxed Forward CTTGGTCGCAGCTTGGGAGTA

Reverse GGACTAACCCTCCTCCCTTTC

CASK KO Forward CTTGGTCGCAGCTTGGGAGTA

Reverse TTTGGGGACTAGATGGGTGTGGTG

Single cell RT-PCR for genotyping

b-Actin 1st Forward TTTGCAGCTCCTTCGTTGCCGGTC

Reverse CCTGGATGGCTACGTACATGG

b-Actin 2nd Forward TTCGTTGCCGGTCCACACCC

Reverse ACATGGCTGGGGTGTTGAAG

CASK 1st Forward CCAGTTTTCAGAACCCTCCA

Reverse GGCTTTCCGTAAGGCTCTCT

CASK 2nd Forward TGTACGAGCTATGCGAGGTG

Reverse CAAAGCCCCCAAGTTTAACA

RT-qPCR

GAPDH Forward CATGGCCTTCCGTGTTCCTA

Reverse CCTGCTTCACCACCTTCTTGA

CASK Forward CTACATGAGACAGATACTGGAA

Reverse CCAAGTTTAACAGGTGCCGAGT

GluN2A Forward CTGAATAAGGACCGGGAATG

Reverse AATGCTGAGGTGGTTGTCATC

GluN2B Forward TGGCTATCCTGCAGCTGTTTG

Reverse TGGCTGCTCATCACCTCATTC
